# Extracellular vesicles derived from nasopharyngeal carcinoma induce the emergence of mature regulatory dendritic cells using a galectin‐9 dependent mechanism

**DOI:** 10.1002/jev2.12390

**Published:** 2023-12-20

**Authors:** Anthony Lefebvre, Camille Trioën, Sarah Renaud, William Laine, Benjamin Hennart, Clément Bouchez, Bertrand Leroux, Delphine Allorge, Jérôme Kluza, Elisabeth Werkmeister, Guillaume Paul Grolez, Nadira Delhem, Olivier Moralès

**Affiliations:** ^1^ Univ. Lille, Inserm, CHU Lille U1189 – ONCO‐THAI – Assisted Laser Therapy and Immunotherapy for Oncology Lille France; ^2^ Univ. Lille, CNRS, Inserm, CHU Lille, UMR9020‐U1277 ‐ CANTHER ‐ Cancer Heterogeneity Plasticity and Resistance to Therapies Lille France; ^3^ CHU Lille, Laboratoire de Toxicologie Lille France; ^4^ Univ. Lille, CNRS, INSERM, CHU Lille, Institut Pasteur de Lille, US 41 – UAR 2014 – PLBS Lille France

**Keywords:** extracellular vesicles, galectin‐9, IDO, IL‐4, IL4I1, mature regulatory dendritic cells, metabolism, nasopharyngeal carcinoma

## Abstract

Nasopharyngeal carcinoma‐derived small extracellular vesicles (NPCSEVs) have an immunosuppressive impact on the tumour microenvironment. In this study, we investigated their influence on the generation of tolerogenic dendritic cells and the potential involvement of the galectin‐9 (Gal9) they carry in this process. We analysed the phenotype and immunosuppressive properties of NPCSEVs and explored the ability of DCs exposed to NPCSEVs (NPCSEV‐DCs) to regulate T cell proliferation. To assess their impact at the pathophysiological level, we performed real‐time fluorescent chemoattraction assays. Finally, we analysed phenotype and immunosuppressive functions of NPCSEV‐DCs using a proprietary anti‐Gal9 neutralising antibody to assess the role of Gal9 in this effect. We described that NPCSEV‐DCs were able to inhibit T cell proliferation despite their mature phenotype. These mature regulatory DCs (mregDCs) have a specific oxidative metabolism and secrete high levels of IL‐4. Chemoattraction assays revealed that NPCSEVs could preferentially recruit NPCSEV‐DCs. Finally, and very interestingly, the reduction of the immunosuppressive function of NPCSEV‐DCs using an anti‐Gal9 antibody clearly suggested an important role for vesicular Gal9 in the induction of mregDCs. These results revealed for the first time that NPCSEVs promote the emergence of mregDCs using a galectin‐9 dependent mechanism and open new perspectives for antitumour immunotherapy targeting NPCSEVs.

## 1 INTRODUCTION

Exosomes are a distinct population of small extracellular vesicles (EVs) from endocytic origin (Mathivanan et al., 2010). They belong to the small EVs compartment with a diameter ranging from 30 to 150 nm. They arise from late endosomal compartments called multivesicular bodies (MVBs) (Théry et al., 2009) and are released into the extracellular microenvironment by fusion of the MVBs with plasma membrane. Exosomes are secreted by most cell types, notably in stress condition, but especially immune cells and tumour cells (Kharaziha et al., 2012). They behave as intercellular mediators by transmitting molecules they convey. Their composition and function may vary depending on the cell of origin, but usually they carry different forms of proteins, lipids, nucleic acids (Valadi et al., 2007) and other substances giving them their capacities (Duvallet et al., 2016; Qazi et al., 2009; Théry et al., 1999).

Nasopharyngeal carcinoma (NPC) is a malignant epithelial tumour of the upper aerodigestive tract able to secrete large amount of immunosuppressive EVs like exosomes (Ng et al., 2020; Wu et al., 2018). Foci of prevalence are essentially found in the Guandong region, in South‐East Asia, Greenland and Northern Maghreb. This distribution indicates multidimensional etiologic factors such as genetic predispositions, the environment, dietary habits and Epstein–Barr virus (EBV) infection which is the main incriminating agent (Bei et al., 2012; Chang & Adami, 2006; Chen et al., 2019). In fact, NPC is associated with a latency II profile infection of EBV in which latent proteins EBNA1, LMP1, LMP2A and LMP2B are expressed and participate in cell cycle dysregulation, transformation and cell survival (Young & Rickinson, 2004). One of the major features of NPC is the presence of an important immune infiltrate within the primary tumour: mostly T cells (LT) with a minority of B lymphocytes (LB), monocytes, dendritic cells (DCs) and eosinophils (Gourzones et al., 2012; Chen et al., 2020; Tang et al., 2001). Nevertheless, this intra‐tumour infiltration of immune cells does not prevent the development of the tumour mass. Indeed, the tumour microenvironment (TME) has been described as highly immunosuppressive in NPC and dominated by immunosuppressive actors notably tumour derived EVs. Actually, tumour‐derived exosomes are produced in large quantities by NPC cells and described by us and others as displaying immunosuppressive properties (Klibi et al., 2009; Mrizak et al., 2015; Ye et al., 2014). They carry specific immunosuppressive molecules such as the EBV protein LMP1 and the galectin‐9 which give NPC exosomes a suppressor role, favourable to tumour immune escape (Keryer‐Bibens et al., 2006). Galectin‐9 (Gal9) is a tandem repeat galectin containing two carbohydrate recognition domains joined by a linker and which have a high affinity for β‐galactoside residues. Gal9 is a lectin mainly known for its immunosuppressive properties notably by inducing apoptosis of T helper Th1 (Zhu et al., 2005), inhibition of Th17 (Seki et al., 2008) and expansion of immunoregulatory populations including macrophages M2 (Zhang et al., 2019), myeloid‐derived suppressor cells (MDSC) (Yamauchi et al., 2005) and Regulatory T cells (Tregs) (Seki et al., 2008; Wu et al., 2014) in particular via its binding to T‐cell immunoglobulin and mucin containing protein‐3 (TIM3). Gal9 expression is mainly associated with poor prognostic in many cancers such as renal cell carcinoma or in acute myeloid leukaemia, pancreatic ductal adenocarcinoma, gastric carcinoma (Chen et al., 2017; Morishita et al., 2021). On the contrary, an increase in Galectin‐9 is associated with a good prognosis in some solid cancers such as colon cancer or hepatocellular carcinoma (Fujita et al., 2015; Morishita et al., 2021; Zhou et al., 2018). This ambivalent effect on tumour prognosis makes its role in tumour progression ambiguous and still debated.

Regulatory T cells represent another major immunosuppressive actor of the TME in NPC. They have been described as the most abundant population of tumour infiltrating lymphocytes (TILs) and at the periphery in NPCs patients where they can represent up to 11%–12% and 8%–2%, respectively (Liu et al., 2021; Sh et al., 2021; Yip et al., 2009). We recently described that NPC‐exosomes were able to recruit and activate Tregs, further promoting the immunosuppressive TME (Mrizak et al., 2015). Tregs are the major players in immune tolerance and are able to regulate the immune response by suppressing effector cells in many well described ways (Shang et al., 2015; Shevach & Thornton, 2014; Thornton & Shevach, 1998). Thus, a large amount of Treg is systematically associated with tumour progression and poor prognosis in most of cancer type (Dons et al., 2012). One of the main cellular mechanism of Treg differentiation in the periphery involves tolerogenic dendritic cells (tDC) also known to contribute to the tumour immune escape (Suciu‐Foca et al., 2009).

DC are myeloid bone‐marrow derived cells with a pivotal role in between innate and adaptive immune response and are central regulators of immune tolerance (Steinman, 2012). At their immature state, they are present in tissues where they act as sentinels to detect, capture and process antigens and respond to danger signals in the periphery, via many of the Pattern Recognition Receptors (PRR) they harbour. This leads to a maturation process during which they will migrate into draining secondary lymphoid nodes (dLN), interact with lymphocytes to present the processed antigen and give rise to an appropriate immune response (Steinman & Idoyaga, 2010). During the maturation process, DC notably increase the expression of their antigen‐presenting molecules, co‐stimulatory molecules to reinforce the immune activation, and C‐C motif chemokine receptor type (CCR)‐7 allowing the migration to dLN (Plesca et al., 2022; Sozzani, 2005). They also undergo metabolic changes with a switch from oxidative phosphorylation (OXPHOS) and fatty acid oxidation (FAO) to glycolytic pathway occurs (Pearce & Everts, 2015). After this process, mature DCs (mDC) become professional antigen presenting cells (APC) able to activate and orientate the immune response properly. However, there are other population of DC able to inhibit the immune response. Tolerogenic dendritic cells (tDC) are the immunosuppressive counterpart of immunostimulatory mature (Steinman et al., 2003). They regulate the immune response and prevent the development of autoimmune diseases by enabling immune peripheral tolerance. They are typically characterized by decrease in the expression of co‐stimulatory molecules, proinflammatory cytokines, but upregulate expression of inhibitory molecules (PD‐L1 and CTLA‐4), expression and activity of metabolic enzymes like IDO and their ability to block effector T cell proliferation whilst enhancing Treg function and differentiation (Kim et al., 2018). Conversely to mDC, tDC retain their oxidative phosphorylation (OXPHOS) and fatty acid oxidation (FAO) metabolism (Sim et al., 2016). In recent years, single‐cell RNA sequencing analysis, have made possible to identify across different DC lineages a common DC program named “mature DCs enriched in immunoregulatory molecules” or mature regulatory DCs (mregDCs) (Kvedaraite & Ginhoux, 2022; Maier et al., 2020; Zilionis et al., 2019). mregDCs are characterized by a signature specific to DC maturation, migration, immunosuppressive molecules (PD‐L1, PD‐L2 and CD200), a lack of IL‐12 expression and production depending on the TME (Maier et al., 2020).

In this study, we expected to evaluate the impact of NPC derived SEVs on the maturation of human monocyte‐derived DC (moDC). Our hypothesis being that in the context of NPC, tumour SEVs favour the emergence of tDC that subsequently contribute to the worsening of NPC by promoting and maintain tumour immune‐escape. After SEVs characterisation, we have evaluated the effect of NPC‐SEVs on DC's (NPCSEV‐DC) function, phenotype and migration. Interestingly, we observe that NPCSEV‐DC exhibit a tolerogenic function with default in T cell activation. Surprisingly, NPCSEVs do not modify DCs’ phenotype but rather their function. In fact, we observed that after the differentiation process NPCSEV‐iDC secrete higher level of IL‐10 and have a strong IDO activity. Interestingly, after maturation state, NPCSEVs give rise to mature regulatory DCs (mregDCs) whom lack IL‐12 and IL‐6 secretion, but produce the immunoregulatory cytokine IL‐4 and exhibit a weak oxidative metabolism. Moreover, we demonstrated that NPCSEV‐DC could be attracted by NPCSEVs. Finally, we explored the involvement of Gal9, in the immunosuppressive effect of SEVs on DC. After validating the effect of recombinant form of Gal9, we performed the same battery of tests in the presence of a neutralizing antibody. Our data confirm that Gal9 conveyed by NPCSEVs participates in the emergence of mregDCs. This description of the interactions between NPCSEVs and DCs is an additional mechanism revealing new insights about how tumour derived SEV mediate immune evasion in NPC context and could represent a novel way for immunotherapeutic approaches.

## 2 MATERIAL AND METHODS

### 2.1 Extracellular vesicles production and characterization

#### 2.1.1 NPC tumour cell line

Patient‐derived EBV‐positive xenografted tumours (C15) were permanently propagated (every 4 to 5 weeks) by subcutaneous passage in SCID mice as previously described (Klibi et al., 2009). In accordance with institutional guidelines, homozygous CB‐17 scid/scid (SCID) mice derived from breeding stocks provided by J.P Decavel (Institut Pasteur de Lille: IPL), were housed under specific pathogen‐free conditions at the animal facility of the IPL (Lille, France) (CEEA152010 Proto 20151). The body mass and the tumour mass and volume of each animal were monitored. Regarding the criteria for tumour volume measurement, we used a caliper to estimate the length and width of the tumour and assumed that the tumour had an ellipsoidal shape. We then used the formula for the volume of an ellipsoid ((4/3)*π* a*b*c) where a, b and c were, respectively, the length, width and height to the centre of the ellipse and considered that the width and height were equivalent, which led to the final used formula ((4/3)*π*(a/2)*(b/2)^2^). The mass of the tumours was measured following their recovery after 4 weeks.

#### 2.1.2 Tumour extracellular vesicles generation

We used a classical dissociation method for the extracted tumour mass. First, a step‐by‐step mechanical dissociation into fresh culture medium (RPMI, Gibco, France) added with gentamycin (10 µg/mL) (Thermo Fisher Scientific, Waltham, MA, USA) followed by decantation into fresh medium to eliminate dead cells and debris. This step was repeated several times until we get very fine pieces of tumour into a clear culture medium. The second step consisted in the enzymatic digestion of the tumour pieces with the addition of collagenase II (2 mg/mL) (Serlabo, Entraigues‐sur‐la‐Sorgue, France) and DNase1 (5 µg/mL) (Sigma–Aldrich, St. Louis, MO, USA) into fresh Petri dishes filled with fresh culture medium added with gentamycin (10 µg/mL). The Petri dishes were incubated at least 3 h at 37°C, 5% CO2, 95% humidity.

The third step consisted in the dissociation of the digested tumour pieces into a single cell suspension by mechanical dissociation. The content of the Petri dishes, after enzymatic digestion, was aspirated with 10 or 5 mL sterile pipet and strongly released into a fresh 50 mL conical tube, the dishes were rinsed with the addition of fresh culture medium to recover all the cells. Then several deep aspiration/release steps were repeated to dissociate all the tumour pieces. At the end of this process, the cell suspension was filtered into a 100 µm nylon cell‐strainer (Corning Incorporated, NY, USA) to eliminate the last non‐dissociated tumour fragments and debris; and centrifuged for 10 min, at 1200 rpm at room temperature. During this time, some fractions of the filtered cell suspension were used to quantify the cell suspension.

The fourth step consisted of the purification and enrichment of the cell suspension in human tumour cells by depleting mouse cells. To this, we used a mouse cell depletion kit (Miltenyi Biotec, Bergisch Gladbach, Germany) that has been designed for the enrichment of untouched human cells upon xenotransplantation. This kit allows the release of leucocytes, red blood cells, platelets, fibroblasts and endothelial cells of mouse origin. We strictly used it following the instruction of the supplier as described in the data sheet. Briefly, total cells were magnetically labelled with a cocktail of monoclonal antibodies conjugated with microbeads. Then, the cell suspension was loaded onto a specific column, which was placed in the magnetic field of a separating device. The magnetically labelled mouse cells were retained within the column. The unlabelled human cells run through.

We then get human tumour cells depleted in mouse cells able to be used for the culture phase to enrich the culture medium in extracellular vesicles. To this, cells were dispatched into 75 cm^2^ culture flask (Sarstedt, Nümbrecht, Germany) 1 million cells (to get 80% confluence) per 1,5 mL of non EVs contaminated culture medium (RPMI, Gentamycin 10 µg/mL, 1.5% of ultracentrifugated (TLA100 Beckman rotor, at 127 811 g, 48 min, +4°C) clarified foetal bovine serum Gibco, Thermo Fisher Scientific, Waltham, MA, USA), and cultured for 48 h at 37°C, 5%CO2 and 95% humidity.

Finally, after 48 h of culture, supernatants were saved and either used directly to start extracellular isolation or cryopreserved into 50 mL tubes at −20°C until further use.

#### 2.1.3 Small extracellular vesicles isolation

Isolation of Small extracellular vesicles (SEVs) from C15 xenograft culture media was adapted from the method described by Mrizak et al. by differential ultracentrifugation and flotation on a D_2_O/sucrose cushion (Mrizak et al., 2015). Fresh or defrosted culture supernatants were centrifuged by three consecutive centrifugations, first at 300 g for 10 min, then at 1900 g for 15 min and finally at 12000 g for 35 min. Then supernatants were clarified by ultracentrifugation at 127 811 g for 48 min using Ti45 Beckman rotor, resulting in a pellet of low‐density vesicles. The clarified supernatants were recovered and frozen for further experiments. SEVs contained in this pellet were further purified on a cushion made of sucrose in deuterium oxide (D_2_O; Sigma–Aldrich Chimie, Lyon, France). The pellet was resuspended in filtered PBS and then loaded on top of a 1 mL D_2_O/sucrose solution (20 mM Tris, 30% sucrose D_2_O, pH 7.4) in a 10 mL polycarbonate tube. This gradient was then subjected to ultracentrifugation at 77 175 g in SW 41 Ti Beckman rotor for 75 min. The cushion containing the SEVs was then collected, diluted in filtered PBS and SEVs were pelleted by ultracentrifugation at 109 655 g in a SW 41 Ti Beckman rotor for 90 min. Two further washing steps were carried out in a smaller volume (ultracentrifugation at 128 405 g using TLA100 Beckman rotor). Isolated SEVs were diluted 1:100 and total protein concentration was quantified according to the manufacturer's instructions (Biorad, Hercules, CA, USA) based on a Bradford dye‐binding method, using Ascent Software v2.06 (Multiskan RC Thermo Labsystems, Thermo Fisher Scientific, Waltham, MA, USA). SEVs were stored in phosphate buffered saline in a frozen container (Mr. Frosty, Nalgene, ThermoScientific, Waltham, MA, USA) at −80°C until further use.

#### 2.1.4 SEVs characterization using electron microscopy

Around 2 µg of SEVs were diluted in PBS and placed on formvar‐coated 200 mesh copper grids rinsed and contrasted with 2% phosphotungstic acid (PTA). Grids were rinsed with PBS once and then fixed with glutaraldehyde and contrasted with 2% PTA. Images were obtained with a Hitachi H7500 transmission electron microscope (TEM) equipped with a wide‐field 1024 × 1024‐pixel digital camera from AMT Advantage HR (Elexience, France). EV size was measured using Fiji/ImageJ software (Version 2.9.0). To do this, the 100 nm scale was measured to obtain the reference length. The diameter of each vesicle was then measured in the same way to obtain different length values using the Fiji software. Using the following cross product: (EV length × 100)/(reference length), we were able to determine the diameter of each of the EVs. Finally, the mean diameter of the EVs and the standard deviation were calculated with Excel software version 16.53 (Microsoft, Redmond, WA, USA).

#### 2.1.5 SEVs characterization by tunable resistive pulse sensing (TRPS)

Size distribution of isolated SEVs were determined by tunable resistive pulse sensing (TRPS) using Exoid and NP100 Nanopore (Izon Bioscience, Lyon, France). Briefly, NP100 Nanopore was coated with different buffers from the reagent kit supplied by Izon. Before each analysis, calibration of NP100 was achieved with calibration beads provided in the kit (CPC100 diluted at 1/1000) tested under three different pressures. SEVs fractions were diluted at 1/500 or 1/1000 in PBS, loaded in the Nanopore and analysed at the same three pressures than calibration beads. The measurement conditions for the sample were as follows: stretch 45.6 mm, a voltage to obtain a current of almost 100 nA. 500 particles and three pressure levels were used for calibration beads and samples analysis. The Exoid control suite software (version V1.0.0.181) was used for data recording and Izon data suite (version V1.0.2.32) was used for calculating nanoparticle size ranges and concentrations.

#### 2.1.6 EV‐TRACK knowledgebase

We have submitted all relevant data of our experiments to the EV‐TRACK knowledgebase (EV‐TRACK ID: EV220302) (Van Deun, Hendrix & EV‐TRACK consortium, 2017).

### 2.2 Western blot analysis

Different cell subsets, clarified culture medium and SEVs were lysed in RIPA buffer consisting of 20 mM Tris‐HCM, 50 mM NaCl, 5 mM EDTA, 1% Triton X‐100, 0.02% sodium azide supplemented by a cocktail of proteases inhibitors (Sigma–Aldrich, St. Louis, MO, USA). After centrifugation (14 000 rpm, 30 min), cell debris were removed and supernatants were collected. Protein concentrations were measured using Bio‐Rad Protein Assay according to manufacturer's instructions (Biorad, Hercules, CA, USA).

Ten microgram of proteins were separated by SDS‐PAGE electrophoresis using gradient pre‐casts gels (NuPAGE NOVEX 4%–12% gradient, Bis‐Tris, Life technology, Carlsbad, CA, USA) in standard conditions except when planning detection of CD63, which require non‐reducing conditions. Then proteins were transferred on PVDF membrane (Immobilon‐P, Sigma–Aldrich, St. Louis, MO, USA). The membrane was blocked for 2 h at room temperature (RT) in casein (2 g/L) blocking buffer, and then incubated overnight at 4°C with primary antibodies [mouse anti‐human‐rat‐mouse HLA‐Drα mAb 1:200 (sc‐53499, Santa Cruz Biotechnology INC., Dallas, TX, USA); mouse anti‐human CD63 mAb 1:1000 (ab59479, Abcam, Cambridge, UK); mouse anti‐human‐mouse TSG‐101 mAb 1:500 (4A10, Abcam, Cambridge, UK); mouse anti‐EBV LMP1 mAb 1:1000 (CS1‐4, Abcam, Cambridge, UK); rabbit anti‐human Calnexin 1:20000 (EPR3632, Abcam, Cambridge, UK); rabbit anti‐human‐mouse‐rat‐chicken Actin 1:5000 (EPR16769, Abcam, Cambridge, UK); rabbit anti‐human IDO 1:400 (D5J4E, Cell Signalling Technology, USA); mouse anti‐cyclophilin B mAb 1:1000 (Cell Signaling Technology, USA) and rabbit anti‐human Galectin‐9‐CT‐L1 mAb 1:400 (GalPharma, Kagawa, Japan)].

Membranes were washed with PBS‐Tween 0.05%, then incubated for 1 h at RT with peroxidase‐conjugated secondary antibodies anti‐mouse or anti‐rabbit, 1:10000 (GE Healthcare, Wauwatosa, USA). Specific protein signals were visualized using Western Lightning Plus‐ECL, Enhanced Chemiluminescence Substrate kit (Thermo Fisher Scientific, Waltham, MA, USA) and read by chemiluminescence with LAS‐3000 (Fujifilm Global, Saint Quantin en Yvelines, France) using the Image Lab v6.1 software (BioRad, Marnes‐la‐Coquette, France).

### 2.3 Human peripheral blood mononuclear cells (PBMCs) isolation

Human blood samples were collected from healthy adult donors with informed consent obtained in accordance with approval of the Institutional Review Board at the Institut de Biologie de Lille (DC‐2020‐3942). Peripheral Blood Mononuclear cells (PBMCs) were isolated from peripheral blood samples by density gradient centrifugation using lymphocyte separation medium (Eurobio, Les Ullis, France) and leucosep (Eurobio, Les Ullis, France) according to the manufacturer's instructions (Dutcher, Brumath, France).

### 2.4 PBMC co‐culture and T lymphocytes proliferation assay

PBMCs were cultivated in ML10 medium made with Roswell Park Memorial Institute (RPMI) 1640 medium (Gibco, ThermoFisher, Waltham, MA, USA) supplemented with sodium pyruvate (1 mM), non‐essential amino acids MEM 1x, HEPES (25 mM), 2‐mercaptoethanol (50 µM), gentamicin (10 µg/mL) (Thermo Fisher Scientific, Waltham, MA, USA), and 10% Human Serum AB (SAB, Sigma–Aldrich, St. Louis, MO, USA). SEVs and recombinant Galectin‐9 immunomodulatory properties were tested by a proliferation assay. C15SEVs (0.5 and 5 µg/mL) and recombinant Galectine‐9S (Gal9S, 3 µg/mL) (Galpharma, Kagawa, Japan) were added to PBMCs (100 000 cells/wells) in round bottom 96‐well plate (Corning Incorporated, NY, USA) for 48 or 72 h. Then the impact of SEVs and recombinant Galectin‐9 blocking were tested by a proliferation assay. C15SEVs (5 µg/mL) and recombinant Galectine‐9S (Gal9S, 3 µg/mL) (Galpharma, Japan) blocked or not with anti‐Gal9 antibody (1g3, 3 µg/mL) (Biotem, Apprieu, France), isotype mouse IgG1K (3 µg/mL) (Biolegend, San Diego, CA, USA) or lactose (5 mM) (Fluka, St. Louis, MO, USA) were added to PBMC (100 000 cells/wells) in round bottom 96‐well plate (Corning Incorporated, NY, USA) for 48 or 72 h. We realized a pre‐incubation of C15SEVs or recombinant Gal9S with 1g3 / isotype / lactose during at least 2 h before adding them to culture with PBMC. The cells were activated with plate‐bound anti‐CD3 mAb (0.5 µg/mL) (Miltenyi Biotec, Bergisch Gladbach, Germany), incubated at 37°C for 1 h before the culture and anti‐CD28 mAb (0.5 µg/mL) (Clinisciences, Montrouge, France) added at the time of the culture. Proliferation was measured after [methyl‐^3^H]‐thymidine (1µCi/well) (PerkinElmer, Courtaboeuf, France) incorporation for the last 18 h before harvesting. Radioactivity was determined using a β‐counter (1450 Trilux, Wallac, Finland). Each proliferation assay was carried out in triplicate and estimated in count per minute (cpm) using Microbeta software.

### 2.5 Human monocyte and T cell isolations

Monocytes and total CD3 T cell were isolated from PBMCs using, respectively, a positive magnetic selection CD14+ and CD3+ isolation kit (Miltenyi Biotec, Bergisch Gladbach, Germany) according to the manufacturer's instructions with an average purity of 95% and 75%, respectively.

### 2.6 Monocytes derived dendritic cells (moDC) generation

Monocytes (10^6^ cells/mL) were cultivated in complete medium (RPMI 1640, Gibco, Thermo Fisher Scientific, Waltham, MA, USA) with 10% clarified FBS (Gibco, Thermo Fisher Scientific, Waltham, MA, USA), 50 µg/mL of gentamycin (Thermo Fisher Scientific, Waltham, MA, USA) in 6‐well plates (Corning Incorporated, NY, USA). To allow monocytes differentiation into immature DCs (iDC), GM‐CSF (25 ng/mL) and IL4 (10 ng/mL) (PeproTech Inc, Rocky Hill, NJ, USA) were added to the medium. Testing condition was generated by adding C15SEVs (C15SEVs) (5 µg/mL), Recombinant galectin‐9S (Gal9S) (3 µg/mL) (Galpharma, Kagawa, Japan), antibody neutralizing galectin‐9 (1g3, 3 µg/mL) (Biotem, Apprieu, France), isotype mouse IgG1K (3 µg/mL) (Biolegend, San Diego, CA, USA) or lactose (5 mM) (Sigma–Aldrich, St. Louis, MO, USA) in culture with monocytes. We realized a pre‐incubation of C15SEVs or recombinant Gal9S with 1g3 / isotype / lactose during at least 2 h before add them to culture with DC. At day 4 dexamethasone (393 ng/mL) (Sigma–Aldrich, St. Louis, MO, USA) was added to tolerogenic control. On day 5, culture medium was removed and centrifuged (300 g, 10 min). The cell pellet was then suspended in clean culture medium and refreshed with new cytokine before added to the corresponding well. The culture media were replaced with new medium containing ± IL1β (10 ng/mL) and TNFα (20 ng/mL) to obtain mature DCs (mDC), and supplemented with dexamethasone (393 ng/mL) (Sigma–Aldrich, St. Louis, MO, USA) and vitamin D3 (39 ng/mL) (Life technology, Carlsbad, CA, USA) to generate tolerogenic DCs (tDC). IL1β (10 ng/mL) and TNFα (20 ng/mL) were also added to test conditions at day 5. Monocytes were maintained in culture to day 5 and served as differentiation control. Cells were recovered and evaluated by flow cytometry for cell surface maturation markers at day 5 and 7. Supernatants recovered at day 5 and 7 of the culture were stored at −80°C until further use. Cells were harvested at day 5 and 7 for further experiments.

### 2.7 Mixed Leukocyte Reaction (MLR)—DC/T cell co‐culture

Functional properties of the DCs were measured by their ability to inhibit the proliferative response of T cell in a Mixed Leukocyte Reaction (MLR) test. CD3+ T cells and monocytes/moDCs were cultured alone (as a control) or together (MLR) for 48, 72 and 96 h with a ratio of 1:10 (DC:CD3+) in ML10 medium supplemented with 10% SAB (Sigma–Aldrich, St. Louis, MO, USA) in a round bottom 96‐well plate (Corning Costar). DCs or monocytes were irradiated at 50 Gy before the culture and T cells were activated with plate‐bound anti‐CD3 mAb (1 µg/mL) (Miltenyi Biotec, Bergisch Gladbach, Germany) and anti‐CD28 mAb (0.1 µg/mL) (Clinisciences, Montrouge, France). T cells proliferation was measured after [methyl‐^3^H] thymidine (1 µCi/well) (PerkinElmer, Courtaboeuf, France) incubation for the last 18 h before harvesting. Radioactivity was determined using a β‐counter (1450 Trilux, Wallac, Finland). Each proliferation assay was carried out in triplicate and estimated in count per minute (cpm) and then normalized on the control condition (CD3+ T cells co‐cultured with iDC or mDC).

### 2.8 Flow cytometry analysis

DCs differentiation and maturation were determined with the analysis of different membrane markers by flow cytometry using Attune NxT (Thermo Fisher Scientific, Waltham, MA, USA). After their harvest, 5.10^5^ cells were taken up in a volume of 100 µL of PBS without Ca2+/Mg2+ (PBS^−/−^) and the fragment crystallizable receptors (FCR) were blocked with a FCR blocking reagent (Miltenyi Biotec, Bergisch Gladbach, Germany) for 10 min at 4°C. Then the cells were labelled for 30 min at 4°C in the dark with fluochrome‐conjugated mAbs: CD14‐Pacific Green, CD11c‐Pacific Blue, CD40 – PE, CD80 – APC, CD83 – PECy7, CD86 – FITC, HLADR—PerCP et DC‐SIGN—APC‐Cy7 (Miltenyi Biotec, Bergisch Gladbach, Germany). For each assay, an unmarked control and the appropriate isotypic control mAbs were used for positive signal setting. Finally, median fluorescence intensity (MFI) data were analysed with FlowJo software (Version 10.8.1, Ashland, OR, USA).

### 2.9 Enzyme‐Linked Immunosorbent Assay (ELISA)

Cytokine detection was carried out on the supernatants of monocytes at day 5 and DCs at days 5 and 7 of culture. Cytokine secretions of Interleukin (IL)−10, Transforming Growth Factor (TGF)‐β1, IL‐12p70, IL‐6 and IL‐4 were determined by the Sandwich ELISA (Enzyme‐Linked ImmunoSorbent Assay) method. Briefly, purified primary antibodies (BD Pharmingen, San Jose, CA, USA) were coated overnight at 4°C in 96‐well maxisorp plates (NUNC, Thermo Fisher Scientific, Waltham, MA, USA).  After washes with PBS‐Tween 0.05% and a non‐specific site blocking with PBS‐BSA 3% (Bovine Serum Albumin, Sigma–Aldrich, St. Louis, MO, USA) for 2 h at RT, culture supernatant was incubated in the plate overnight at 4°C. After washes (PBS‐Tween 0.05%), anti‐cytokine biotinylated detection secondary Ab (1 µg/mL) (BD Pharmingen, San Jose, CA, USA), was incubated for 90 min at RT. Followed by washes, the reaction was amplified by adding streptavidin‐peroxidase to 1/10000^th^ (Interchim, Montluçon, France) for 30 min at RT. The plates were revealed by the addition of a solution of H_2_O_2_ (1/1000^th^) and Ortho‐phenylenediamine Dihydrochloride (OPD) at 10 mg/mL (Sigma–Aldrich, St. Louis, MO, USA) in development buffer. This reaction was stopped by addition of HCl 2N (VWR, USA). In parallel we measured the production of the IL4I1 enzyme by the different cells with the Human IL4I1 DuoSet kit following the supplier's recommendations (R&D SYSTEMS). The plates were then read at 492 nm on the spectrophotometer (Multiskan EX, ThermoLabsystems, France) using Ascent Software v2.06. Table 1 shows all the purified and biotinylated antibodies used for ELISA experiments.

**TABLE 1:** Purified and biotinylated antibodies used in ELISA.

	Purified antibodies	Biotinylated antibodies
Anti‐IL‐10	Rat IgG1	Rat IgG2a
Anti‐TGFβ	Rat IgG2a	Rat IgG2a
Anti‐IL‐4	Mouse IgG2a	Rat IgG1
Anti‐IL‐6	Rat IgG1	Rat IgG2a
Anti‐IL‐12p70	Rat IgG2b	Mouse IgG1
Anti‐IL4I1	Mouse anti‐IL4I1	Rat anti‐IL4I1

### 2.10 High performance liquid chromatography (HPLC)

The substrate and product, that is, L‐Tryptophan (Trp) and Kynurenine (Kyn) of IDO (Indoleamine 2,3‐dioxygenase) were quantified in the supernatant of all the culture conditions at day 5 and 7. Kynurenic acid, indole acetic acid and indole lactic acid were quantified in the supernatant of all the culture conditions at day 7. Supernatants were immediately frozen at −80°C for further analysis. Samples were tested by the Toxicology Unit at University Hospital of Lille, France. Concentrations of tryptophan and kynurenin were assayed using an analytical procedure based on electrospray ionization liquid chromatography‐tandem mass spectrometry (LC‐ESI/MS/MS). This procedure was developed according to previously published methods, with slight modifications (W. Zhu et al., 2011). One hundred microliter of supernatants or culture medium were analysed after the addition of 100 µL acetonitrile containing tryptophane‐D5 at 50,000 nM, as an internal standard. The samples were mixed and centrifuged and the supernatant (100 µL) was added to deionized water (500 µL). Fifteen microliters of this mixture were injected onto an UPLC‐MS/MS system (Xevo TQ‐S Detector, Waters, Milford, USA) equipped with an Acquity HSS C18 column (Waters, Milford, USA). Ions of each analysed compound were detected in a positive ion mode using multiple reaction monitoring. MassLinks software (Waters) was used for data acquisition and processing. Results were expressed in nM and Kyn/Trp ratio were calculated and normalized on control condition (iDC for day 5 and mDC for day 7).

### 2.11 Oxygen consumption rate (OCR) measurement

Mitochondria oxygen consumption rate (OCR, O2 mpH/min) of moDCs were measured using Seahorse XFe96 analyser (Seahorse Bioscience, Billerica, MA, USA) with the Wave software (version 2.6.0). Seahorse XFe96 microplates were precoated with poly‐L‐lysine few hours before the experiment. Cells were harvested, washed and suspended in OXPHOS medium (DMEM base, 25 mM glucose, 1 mM pyruvate, 2 mM L‐glutamine [pH 7.35]). Cells (60 × 103/well) were plated in poly‐D‐lysine microplates centrifuged two time at low speed (160 g for 1 min), and incubated in a non‐CO_2_ incubator 1 h at 37°C. Before each OCR measurement, calibration and equilibration of the Seahorse analysers were performed. Each port was loaded with 20 µL of inhibitors diluted in OXPHOS medium. The OCR was performed with all moDC types simultaneously at baseline and after injection of following inhibitors: oligomycin A (2 µM) for mitochondrial complex V inhibition, FCCP_1_ (carbonyl cyanide 4‐(trifluoromethoxy) phenylhydrazone, 0,5 µM) FCCP_2_ (0,75 µM) for maximal respiration induction, rotenone and anti‐mycine A (1 µM each) for electron transportation chain inhibition (Sigma–Aldrich, St. Louis, MO, USA).

### 2.12 In vitro moDCs real‐time chemoattraction assay

For cell chemoattraction experiments, mDC, tDC and C15SEV‐DC, respectively, labelled with Vybrant Multicolor Cell labeling kit DiO, DiD and DiI fluorochrome (Thermofisher Scientific, Waltham, MA, USA) were suspended in RPMI 1640 (Gibco, Thermo Fisher Scientific, Waltham, MA, USA), MEM 10X (Gibco, Thermo Fisher Scientific, Waltham, MA, USA), NaOH 1M, NaHCO3 7.5%, mixed with 1.5 mg/mL collagen I (PureCol, Advanced Biomatrix, San Diego, CA) at the final density of 18 × 10^6^ cells/mL and placed in the migration channels of a μ‐Slide chemotaxis^3D^ chamber (Ibidi, Munich, Germany), in accordance with manufacturer's instructions. The chambers at the left and right side of the migration channels were filled with RPMI 1640, CCL19 (Peprotech, London, UK) (100 ng/mL) or C15SEVs (5 µg/mL) were next added only to the right chamber to generate a chemotactic gradient.

Time‐lapse microscopy was realized using the Zeiss video‐microscope system (Zeiss Axio observer Z1) equipped with a 10x phase contrast objective with (green, red and far‐red laser). The μ‐Slide chemotaxis^3D^ chambers loaded with moDCs were incubated into the 37°C heating stage of the Zeiss system with 5% of CO_2_ and images were taken at a rate of one frame per 5 min for 4–6 h with Zen software (Zen blue v2.3). The Imaris software (Oxford Instrument, USA, V 9.8.0) was used to perform cell tracking and migration movies of mDC, tDC and C15SEV‐DC under C15SEV gradient (Supplementary data 1). For each independent experiment, at least 40–50 cells were monitored excluding immobile cells. Then, MATLAB R2020a software (MathWorks, Natick, M, USA) was used to performed different analysis, graphs and statistical tests. Exported tables from manual tracking from Imaris Software were next put into Excel. The cell tracks were all extrapolated to (x, y) = 0 at time point 0 h. Quantitative analysis of moDC migration was performed by measuring several parameters of the trajectories that give information's on how fast, straight cells move, and how much trajectory is directed toward the chemotactic gradient. We measured the centre of mass (COM), velocity, forward migration indices (FMI) parallel and perpendicular to the gradient, the directness and Rayleigh test as described by Ruez et al. (2018, 43).

### 2.13 Real‐time quantification polymerase chain reaction assays (RTQPCR)

#### 2.13.1 mRNA extraction

Total RNA from cultured cells (1.10^6^ cells) was extracted using the Trizol reagent (Life Technologies, Carlsbad, CA, USA) method according to the manufacturer's instructions. Briefly, 1.10^6^ cells were suspended in 1 mL of Trizol and stored at −80°C until further use. For RNA isolation, 200 µL of Chloroform were added to samples, then cells were centrifuged at 12,000 g for 15 min at 4°C. The upper transparent phase is taken up and total RNA is precipitated with 500 µL of isopropanol and stored at −80°C for 10 min. Then, RNA were centrifuged at 12,000 g for 15 min at 4°C. and the pellet of total RNA was washed with ethanol 70% which was then discarded and samples were left to dry at room temperature. Then samples were then centrifuged at 7500 g, 15 min at 4°C and the pellet suspended in 30 µL of RNAse and DNAse free water (Life Technologies, Carlsbad, CA, USA). RNA concentration and purity were measured by spectrophotometric methods using the NanoDrop (Thermo Scientific, USA). Total RNA was stored at −80°C until further use.

#### 2.13.2 mRNA reverse transcription

Two microgram of total RNA were supplemented with 5 µL of a master mix: 1 µL oligo dT (8 nmol) (Roche Diagnostic, Meylan, France), 4 µL of RNAse/DNAse free water and 0.1 µL RNAsin (40U/µL, Promega, charbonnières, France). Then, samples were incubated at 70°C for 10 min, followed by 5 min at room temperature. After this 10 µL of the reaction mix were added to samples: 6 µL buffer 5X (Tris‐HCL, KCL, MgCL2) (Invitrogen, UK) + 1 µL DiThioThreitol (DTT, 0.1 M) (Invitrogen, UK) + 2 µL deoxyribose Nucleotide Triphosphates (dNTPs, 10 mM) (Amersham Biosciences, UK) + 0.1 µL RNAsin (40 U/µL, Promega, charbonnières, France) + 1 µL Transcriptase Reverse Superscript (200 U/µL, life technologies, Carlsbad, CA, USA). Samples were first incubated at 45°C for 60 min, followed by a second incubation of 5 min at 95°C. Finally, ultrapure distilled water (GIBCO‐Life Technologies) was added to obtain a final concentration of 10 ng total complementary DNA (cDNA)/µL and stored at −80°C until further use.

#### 2.13.3 Aria Mx real time PCR

Transcripts were quantified using real‐time quantitative RT‐PCR with the Aria Mx system (Agilent technologies, France), in PCR‐96‐LP‐FLT plates (Corning Axygen, Mexico). In each well, 10 µL of a specific couple of primers (Eurogentec, Seraing, Belgium) and 1 µL of cDNA sample (equivalent to 10 ng of RNA/µL). PCR reaction were performed according to the manufacturer's instructions, in a final volume of 20 µL, using 2X MESA GREEN qPCR MasterMix Plus for SYBR 258 Assay (Eurogentec, Seraing, Belgium). The pCR program included initial denaturation for 5 min at 95°C, followed by 40 standard amplification cycles as followed: 15 s at 95°C (denaturation) then 1 min at 60°C (annealing and elongation). Fluorescent products were detected at the last step of each cycle.

#### 2.13.4 Data expression

Quantitative PCR reactions were used to quantify gene expression of related DC immunosuppressive activity or effector activity. The housekeeping genes: Glyceraldehyde‐3‐Phosphate DeHydrogenase (GAPDH), Hypoxanthine guanine PhosphoRibosyl Transferase (HPRT) and 18S RNA were used as controls. All primers were designed for real‐time PCR (Table 2) and purchased from (Eurogentec, Seraing, Belgium) or (Sigma–Aldrich, St. Louis, MO, USA). Quantitative analysis was achieved based on the cycle threshold, results were expressed in 2^‐∆∆CT leading to arbitrary value of 1 for the reference group for histograms (Livak & Schmittgen, 2001). Results were expressed in log2 (2^‐∆∆CT) leading to arbitrary value of 0 for the reference group for Heatmaps. Value of each well was calculated using Agilent Aria Mx software V.1.71.

**TABLE 2:** Genes and corresponding primer sequence (forward and reverse) used for RT‐qPCR.

Gene name	HGNC symbol	Forward primer	Reverse primer
CCR7	CCR7	GAGACAACACCACAGTGGACTACAC	GTCAACACGACCAGCCCATT
CD36	CD36	TTGGTTTCTGGGTGGCCAATTCA	TGGGATGATGGTGTTTCCCCCG
DC‐LAMP	DC‐LAMP	CTGGGCGGGCTTTTCGGCTG	TTCTGCAGCGTGCGGCGAAG
FOXO1	FOXO1	CCGACCTCATCACCAAGGC	TTATGACGAATTGAATTCTTCC
GLUT1	GLUT1	CTTCACTGTCGTGTCGCTGT	CCAGGACCCACTTCAAAGAA
STING	STING	ACTGTGGGGTGCCTGATAAC	TGGCAAACAAAGTCTGCAAG
cGAS	cGAS	GCGGTTTTGGAGAAGTTGAA	TGAATTCTGGGGACTTCCAG
GILZ	TSC22D3	CCGAAATGTATCAGACCCCCA	AACGGAAACCACATCCCCTC
IDO	IDO	AAGGTGATGCTGGCCTGCGG	TGGCTGCTGGCTTGCAGGAAT
ILT3	LILRB4	CCCATGGGACATGAGTAGCC	AGCACTTCTCTGCGATGACG
ILT4	LILRB2	GATGCCCCACTCCGTCTAAG	AGTTGAGTGAGCCGTAGCAC
PD‐L1	CD274	AGCCTGGAATTGCAGCTTCA	AAGTTGCATTCCAGGGTCACAT
PD‐L2	PDCD1LG2	TTTATATTCATGACCTACTGGCATTTG	AATTGTCATATTGCTACCATACTCTACCA
TIM‐3	HAVCR2	GCCTTCTGCAACTCCGACA	GGGCATCTTGGTGAAGCCT
Galectin‐9	Galectin‐9	GTGGGTGTGAAAGGCAGCGGT	TCCCAGAAAAGGGGACAGCTGGA
CCR6	CCR6	CCATTCTGGGCAGTGAGTCA	TTTAGCAACTTGCACGTGGC
PVR	PVR	ACTCAGGCATGTCCCGTAAC	GTCTGTGGATCCTGGGAAGA
SOCS3	SOCS3	TCTGTCGGAAGACCGTCAAC	CCTTAAAGCGGGGCATCGTA
NRP1	NRP1	AGCAAGCGCAAGGCTAAGTC	ATCCTGATGAACCTTGTGGAGAGA
SEMA4A	SEMA4A	TCTGCTCCTGAGTGGTGATG	AAACCAGGACACGGATGAAG
IRF4	IRF4	AATCCTCGTGAAGGAGCTGA	ATCCTGCTCTGGCACAGTCT
MAfB	MAfB	AGACGCCTACAAGGTCAAGTGC	CGACTCACAGAAAGAACTCGGG
IL4I1	IL4I1	ATCTCCCACCGAGAGTCATGG	GGGCTTCAGGGTCCGATTGA

### 2.14 Statistical analysis

Graphpad Prism 8.0 software (Graph Pad Software Inc., San Diego, CA, USA) was used for data treatment and statistical analysis. Statistical differences between conditions were determined using ordinary one‐way ANOVA with Tukey's post‐test or Kruskal–Wallis test with Dunn's post‐test after identification and exclusion of outlier's values and Shapiro–Wilk tests. Significance of *p* values are as following *p* ≤ 0.05 (*), *p* ≤ 0.01 (**), *p* ≤ 0.001 (***), and *p* ≤ 0.0001 (****) with *p* < 0.05 being considered statistically significant and highly significant for the others. All *p*‐values have been collected in a single folder with multiple sheet disposable online.

## 3 RESULTS

### 3.1 Small extracellular vesicles (SEVs) and monocytes derived dendritic cells (moDCs) characterization

To produce tumour extracellular vesicles (EVs), we used the C15 NPC cell line which is continuously xenotransplanted into SCID mice. Both mouse mass and tumour volume were monitored over time (Supplementary data 2). Tumour mass, cell quantities after tumour digestion, protein and particle content of EVs were also collected (Table S1).

**FIGURE 1:**
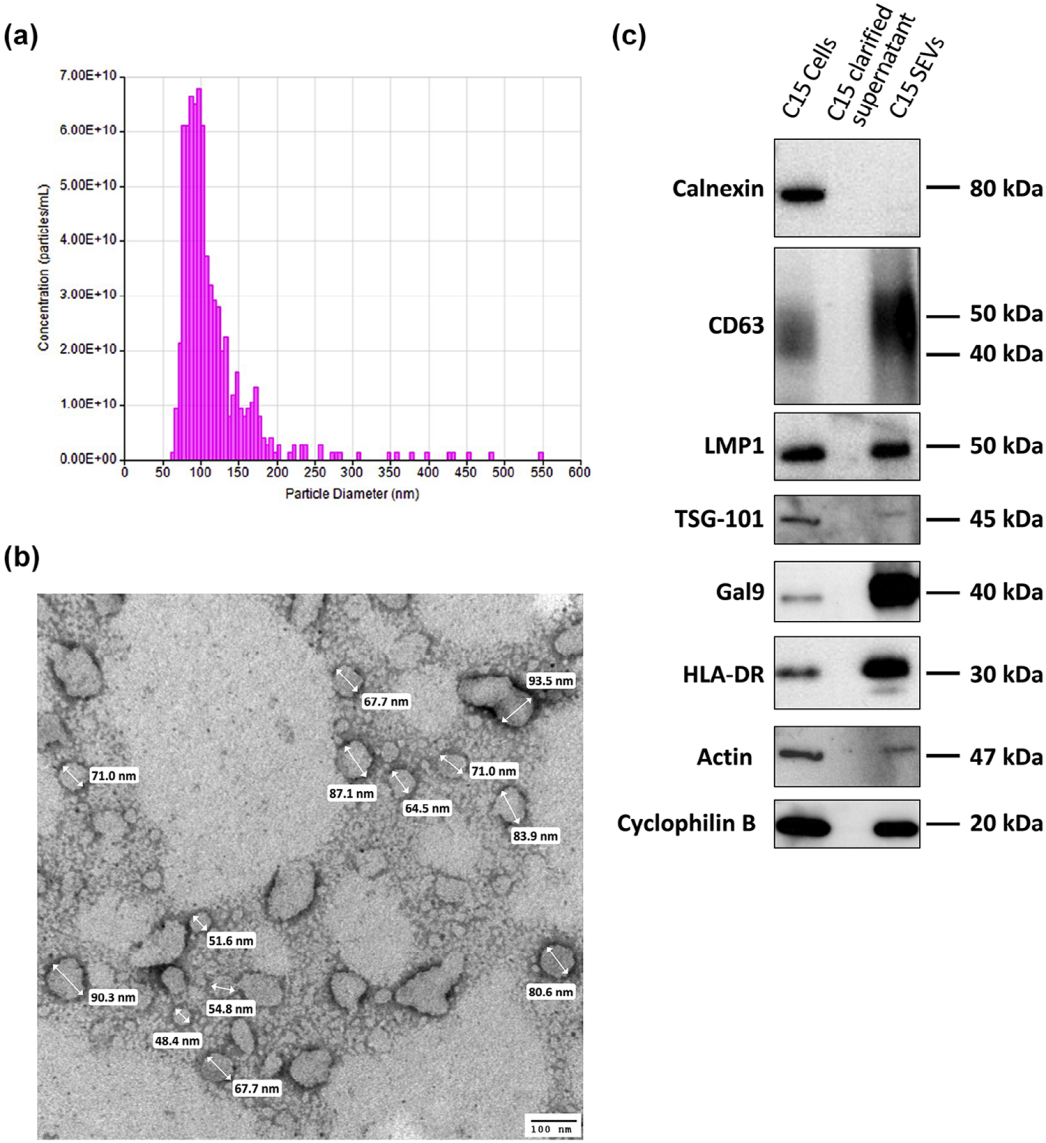
Characterization of biological material small extracellular vesicles (SEVs) and monocytes derived dendritic cells. Morphological analysis of C15 extracellular vesicles (EVs) by (a) TRPS analysis size and concentration. (b) Electron microscopy analysis of C15 EVs suspension contrasted with 2% Phosphotungstic acid (PTA). Scale bar (100 nm) is shown in the lower right corner. (c) Representative graph of western blotted protein markers (Calnexin, CD63, TSG‐101, LMP1, Galectin‐9 (Gal9), HLA‐DR Actin and Cyclophilin‐B) on C15 cells, C15 clarified supernatant and C15SEVs.

To verify that isolated EVs belong to the small EVs (SEVs), we analysed their morphological and phenotypical properties (Figure 1). First, we measured EVs size by Tunable Resistive Pulse Sensing (TRPS) technology (Figure 1a). The samples of EVs isolated from C15 tumour cell supernatant (C15 EVs) are in between 60 and 200 nm in size. Using TRPS analysis, EVs have an average size of 99 nm (±52.57 nm) and a median size of 72.8 nm (±9.13 nm). EVs were also analysed by electron microscopy after PTA contrasting (Figure 1b) and a mean diameter of 71.7 nm (±14.7 nm) has also been recovered showing that as expected isolated EVs belong to the SEVs.

Then, using western blotting we verified that SEVs express some appropriate exosomes markers (Figure 1c). Common exosomal markers such as CD63, HLA‐DR were found enriched, with HLA‐DR highly conveyed on C15EVs. Moreover, we found the presence of the endosomal marker TSG‐101 and the absence of Calnexin (transmembrane marker) as expected for exosomes. Additionally, we looked at the presence of NPC SEVs markers such as LMP1 and Galectin‐9 and those two proteins were present on C15 SEVs. Finally, thanks to the absence of Calnexin, and thanks to the loading control used (Actin and Cyclophilin B) we can confirm that the EVs isolation was sufficient to remove any cellular contamination. Despite of the expression of some endosomal markers (HLA‐DR) and in accordance to the MISEV2018 (Théry et al., 2018), the extracted vesicles will be continuously referred as C15SEVs.

After C15SEVs characterization, we focused on characterization of moDCs. Briefly, moDCs were generated from human monocytes. In order to differentiate monocytes into immature dendritic cells (iDC), we classically added GM‐CSF and IL‐4 to the culture during 5 days. By flow cytometry, cells were first gated for size and granularity followed by doublet exclusion. Then, cells were analysed for CD11c and CD14 expression and we analysed expression of markers in CD11c+ CD14+ (Q2) cells for monocytes, CD11c+ CD14‐ (Q3) cells for moDCs and CD11c‐ CD14‐ (Q4) for unlabelled or isotype mabs (Supplementary data 3A). To attest the proper differentiation of monocytes, we analysed by flow cytometry the dual expression of the membrane markers CD11c, CD14 (Supplementary data 4A.a) and CD11c, DC‐SIGN (Supplementary data 4A.b) and statistical analysis are shown in figure 3. The results indicate that both monocytes and iDCs express CD11c but in contrast to monocytes, iDCs no longer express CD14 and strongly express the lectin DC‐SIGN that confirms the correct differentiation of monocytes into iDC.

Then iDC were matured into control mature DC (mDC) by addition of TNF‐α and IL‐1ß or into control tolerogenic DC (tDC) with dexamethasone/vitamin D_3_ + TNF‐α and IL‐1ß; and analysed by flow cytometry (Supplementary data 4B and C, statistical analysis are shown in Figure 3). As for the differentiation analysis, cells were first selected after granularity and size gating, followed by doublet exclusion. Then cells were analysed for their CD11c and CD14 expression (Supplementary Data 3B). Cytometry analysis of iDC and mDC (Supplementary data 4B) show that both iDC and mDC express CD11c but not the CD14 as expected. Moreover, mDC express more maturation markers CD40, CD80, CD83, CD86, HLA‐DR and less DC‐SIGN associated with immature state than iDC. Same analysis was performed between mDC and tDC (Supplementary data 4C). Both mDC and tDC express CD11c but only tDC express the CD14. Concerning maturation markers, we can observe that tDC express less all‐maturation markers compare to mDC and have the same expression for DC‐SIGN confirming a tolerogenic phenotype.

### 3.2 Immunosuppressive properties of NPC tumour SEVs and tolerogenic effect of C15SEV‐DC

**FIGURE 2:**
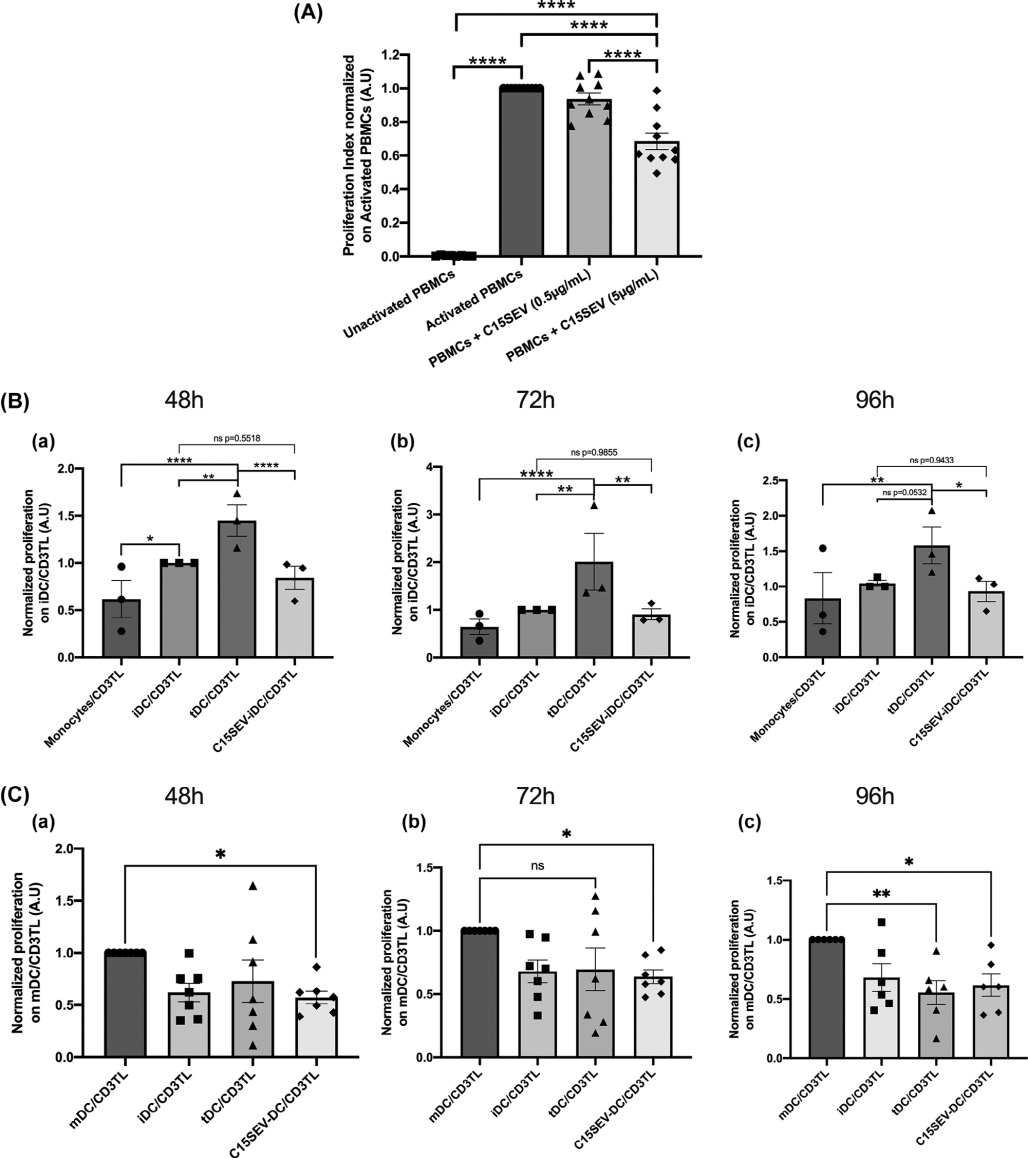
Immunoregulatory properties of C15SEVs and suppressive function of irradiated DCs pre‐cultured with extracellular vesicles. (a) Functional assay of C15SEVs co‐cultured with human PBMCs. The immunosuppressive function of C15SEVs were assessed in a suppression assay. Histograms are representative of ten independent experiments and are expressed in proliferation index normalized on activated PBMCs, MEAN ± SEM. Statistical differences between activated PBMCs and other conditions were analysed by one‐way ANOVA, ****p < 0.0001. (b) Suppression assay of CD3+ T cells co‐cultured for 48 h (B.a), 72 h (B.b) or 96 h (B.c) with irradiated monocytes, immature (iDC), tolerogenic (tDC) and co‐cultured with C15SEVs (C15SEV‐iDC) moDCs after differentiation. Results are expressed in proliferation index normalized on iDC/CD3TL proliferation from three independent experiments, MEAN ± SEM. Statistical differences between conditions were analysed by one‐way ANOVA after outlier identification. *p < 0.05, **p < 0.01, ***p < 0.001, ****p < 0.0001. (c) Suppression assay of CD3+ T cells co‐cultured for 48 h (B.a), 72 h (B.b) or 96 h (B.c) with irradiated mature (mDC), immature (iDC), tolerogenic (tDC) and co‐cultured with C15SEVs (C15SEV‐DC) moDCs after maturation. Results are expressed in proliferation index normalized on mDC/CD3TL proliferation from seven (48 and 72 h) and six (96 h) independent experiments, MEAN ± SEM. Statistical differences between conditions were analysed by one‐way ANOVA after outlier identification. *p < 0.05, **p < 0.01.

NPC SEVs are known to be immunosuppressive decreasing activation and proliferation of immune cells. Thus, we tested the effect of the C15SEVs on human PBMCs proliferation (Figure 2a). Human PBMCs were activated with anti‐CD3 and anti‐CD28 mAbs to mimic a T cell activation and co‐culture with C15SEVs at 0.5 and 5 µg/mL during 72 h and tested for a T Lymphocyte proliferation assay. We observe that the proliferation of human T Lymphocytes is indeed significantly reduced by 30% in the presence of C15SEVs at 5 µg/mL (Figure 2a) confirming their immunosuppressive properties.

Monocytes were differentiated in DC for 5 days by adding GM‐CSF and IL‐4 with or without C15SEV (C15SEV‐iDC). Their maturation was induced in order to get immature DC (iDC, untreated), mature DC (mDC, TNF‐α and IL‐1ß‐treated), tolerogenic DC (tDC, dexamethasone/vitamin D_3_ + TNF‐α and IL‐1ß‐treated) and C15SEV‐DC (C15SEV + TNF‐α and IL‐1ß‐treated moDCs). We then focused on their functional properties after 5 and 7 days of culture. To evaluate the effector or suppressive status of moDCs after differentiation day 5 (monocytes, iDC, tDC and C15SEV‐iDC) and after maturation day 7 (iDC, mDC, tDC C15SEV‐DC) their suppressive potential was tested. For this, we put monocytes/moDCs in co‐culture with total heterologous CD3+ T lymphocytes (CD3+ T cells) and we measured CD3+ T cells proliferation after 48, 72 and 96 h (Figure 2b and c). After differentiation, the proliferation assays show that after 48 h, iDC induce slightly more CD3TL proliferation than monocytes (Figure 2b.a) and a little more proliferation than CD3TL alone (Supplementary data 5a‐c). However, after 72 and 96 h iDCs and monocytes induce similar CD3TL proliferation (Figure 2b.b and c). Interestingly C15SEV‐iDC lead to similar CD3TL proliferation compared to iDC control and less proliferation compared to tDC (Figure 2b.a to c), showing that C15SEV‐iDC keep their immature state and are not able to activate T cells. After maturation, the proliferation assays illustrate that after 48, 72 and 96 h of co‐culture, mDC increase CD3+ T cells proliferation compared to iDC and tDC control (Figure 2c.a, b and c) in a specific manner as compared to controls (Supplementary data 5d‐f). Interestingly, C15SEV‐DC significantly decreased T cells proliferation after 48 h by 42.8% compared to mDC. This decreased proliferation was maintained after 72 and 96 h with, respectively, 36.3% and 38.4% less proliferation compared to the proliferation rate with mDC controls co‐culture (Figure 2c.a, b and c), showing for the first time a functional tolerogenic properties of these C15SEV‐DC.

### 3.3 NPC tumour SEVs do not affect moDC differentiation and maturation phenotype

**FIGURE 3:**
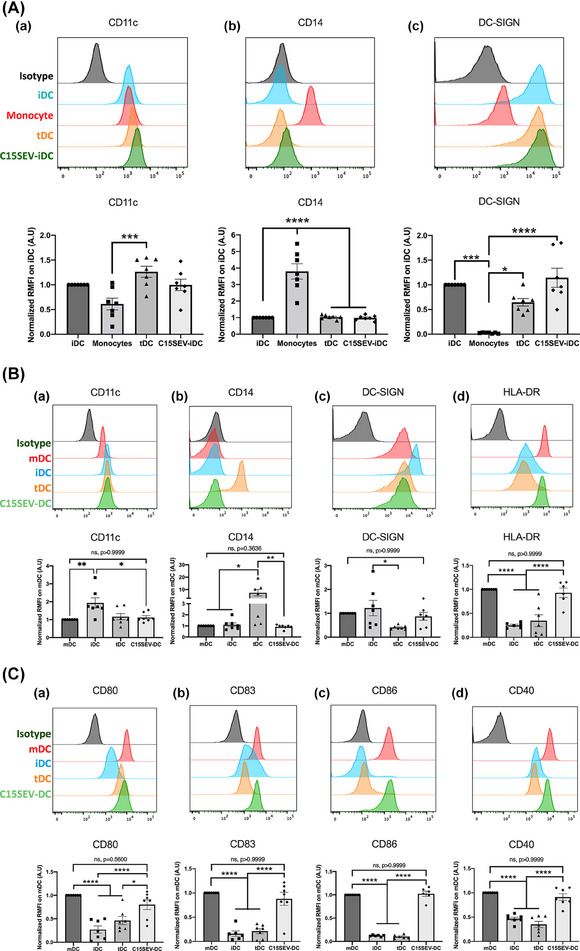
**Phenotypic characterization of moDCs pre‐cultured with SEVs. (A)** Expression level of surface markers CD11c **(a)**, CD14 **(b)**, and DC‐SIGN **(c)** in immature DCs (iDC, blue), monocytes (red), tolerogenic DCs (tDC, orange) and DCs co‐cultured with C15SEV (C15SEV‐iDC, green) after differentiation. Isotype controls are shown in black. The histograms are representative of seven independent experiments and are expressed as the median fluorescence ratio normalized to iDC; MEAN ± SEM. **(B‐C)** Expression level of surface markers CD11c, CD14, DC‐SIGN, HLA‐DR **(B.a‐d)**, CD40, CD80, CD83 and CD86 **(C.a‐d)** in mature (mDC) (Red), immature (iDC, Blue), tolerogenic (tDC, Orange) and co‐cultured with C15SEVs (C15SEV‐DC, Green) moDCs after maturation. Isotype controls are shown in black. The histograms are representative of seven independent experiments and are expressed as the median fluorescence ratio normalized to mDC; MEAN ± SEM. Statistical differences were analysed by one‐way ANOVA after outlier identification. *p < 0.05, **p < 0.01, ***p < 0.001, ****p < 0.0001.

In order to determine if functional tolerogenic property of DC was due to phenotypic modifications, we performed a phenotypic study by flow cytometry analysis of DC membrane markers after differentiation (Figure 3a) and maturation (Figure 3b.C.) process. After differentiation (day 5), we compared the expression of DC population markers CD11c, CD14 and DC‐SIGN (iDC markers) between monocytes and moDCs (iDC, tDC and C15SEV‐iDC) (Figure 3a). Analysis of markers expression level showed that, control DC (iDC and tDC) were well differentiated with loss of CD14 expression and increase of DC‐SIGN (respectively, 41 and 27‐fold more for iDC and tDC) compared to monocytes (Figure 3a.a, b and c). Moreover, when we analysed markers expression in between iDC, tDC and C15SEV‐iDC after differentiation, we do not observe statistically significant differences.

Then after maturation process, iDC, mDC, tDC and C15SEV‐DC were analysed for their respective expression of moDCs markers (Figure 3b.C.). Analysis of DC co‐stimulation and antigenic presentation markers expression indicated that mDC express much more CD40, CD80, CD83, CD86 and HLA‐DR than iDC (respectively, 54%, 73%, 84% 88% and 75% more) confirming their mature state (Figure 3b.d and 3C.a‐d). Similarly, tDC express significantly less all costimulation markers analysed (Figure 3b.d and 3C.a‐d) and present a similar profile to iDC, with the exception of DC‐SIGN, which is, expressed twice as little. All moDCs express similar level of CD11c except for iDC that has a twofold expression compared to the other DCs.

Surprisingly but interestingly, when we compared surface markers expression of C15SEV‐DC to that of mDC, we did not observe significant modification of the surface markers expression (Figure 3b.a‐d and 3C.a‐d), suggesting the emergence of mature regulatory DC.

### 3.4 NPC tumour promote immunosuppression properties in moDCs during differentiation

**FIGURE 4:**
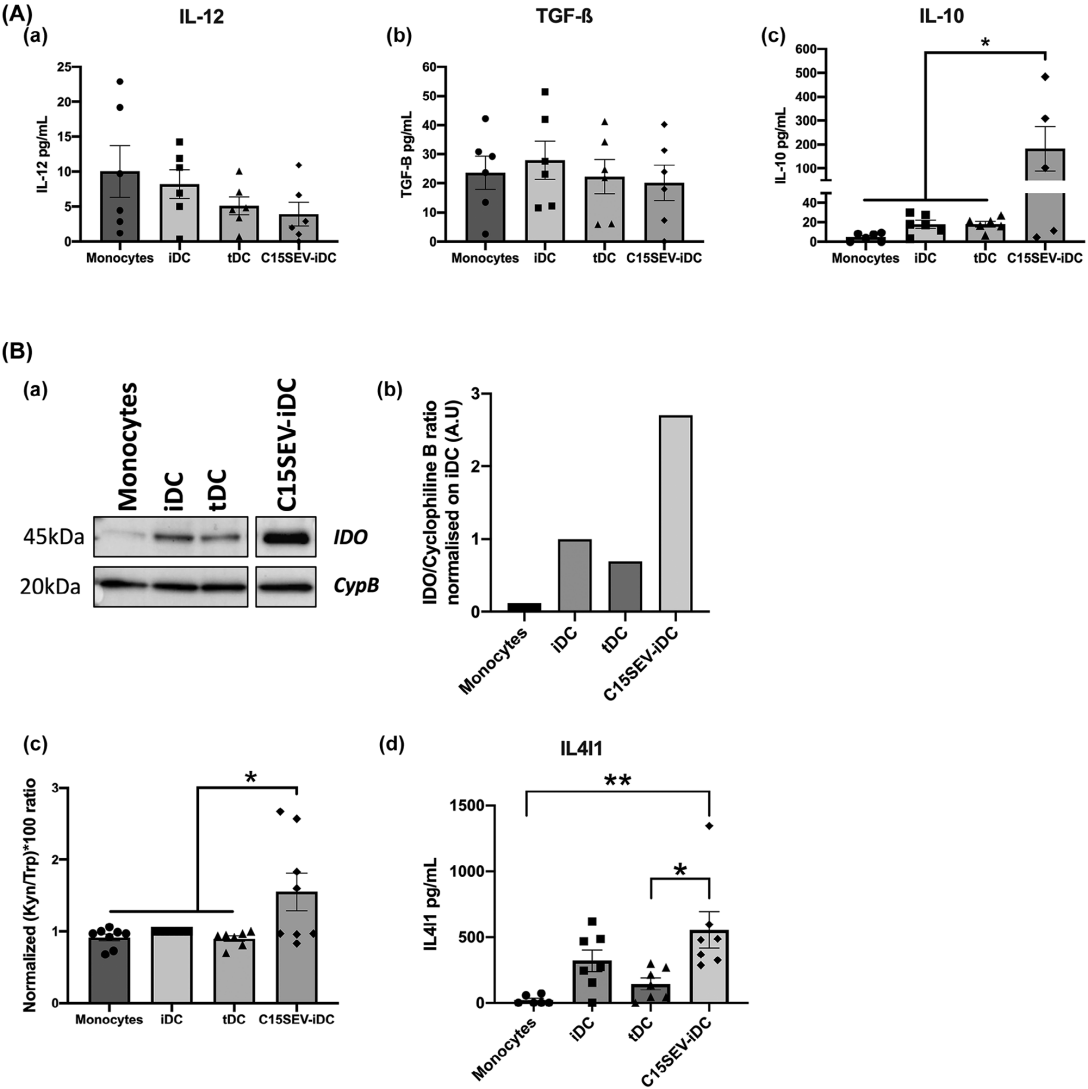
**Immunoregulatory properties of moDCs after differentiation. (A)** Dosage of cytokines secretion by ELISA in the culture supernatant of monocytes and moDCs. **(A.a‐c)** Secretion of the effector cytokine IL‐12 and the suppressive cytokines TGF‐ß and IL‐10. Results expressed in cytokines concentrations (pg/mL) from six independent experiments, MEAN ± SEM. Statistical differences between conditions were analysed by one‐way ANOVA after outlier identification. *p < 0.05 **(B)** IDO expression on DCs and HPLC dosage of Tryptophane metabolites. **(B.a)** Testing of IDO expression by Western blotting were carried out. **(B.b)** The intensities of the IDO bands were quantified via ImageJ software and normalized to the Cyclophilin B (CypB) control and then normalized to the iDC control. **(B.c)** HPLC dosage of Tryptophane and Kynurenine. The histograms are representative of eight independent experiments and are expressed in (Kyn/Trp) ×100 ratio normalized on iDC; MEAN ± SEM. Statistical differences between conditions were analysed by one‐way ANOVA after outlier identification. *p < 0.05, **p < 0.01. **(B.d)** ELISA dosage of IL4I1 in monocytes and moDCs. Representative histogram of six independent experiments, mean ± SEM. Statistical differences between conditions were analysed by one‐way ANOVA after outlier identification. *p < 0.05, **p < 0.01.

In order to understand the biological mechanisms, by which C15SEV‐DC obtain their tolerogenic function, cytokine secretion of moDCs and indoleamine 2,3 dioxygenases (IDO) activity were monitored after differentiation (Figure 4). Effector IL‐12p70 and regulatory cytokines TGF‐β and IL‐10 were studied by ELISA (Figure 4a.a‐c). Concerning effector cytokine, we observed a weak secretion of IL‐12p70 (between 3.9 and 10 pg/mL) by cells after differentiation conditions (Figure 4a.a). About Immunosuppressive cytokines, TGF‐ß is weakly secreted by all cell types (approximately 20 pg/mL) (Figure 4a.b). Interestingly, C15SEV‐iDC shows a significant increase of IL‐10 secretion compared to other cells after differentiation (approximatively 10‐fold more) (Figure 4a.c).

Another characteristic of tDC is their expression and the activity of the immuno‐regulatory enzyme IDO. For this, IDO expression was determined in cultured moDCs by Western Blot and the biologic activity of this enzyme was assessed by HPLC dosage of its substrate and products (Tryptophan and Kynurenine, respectively) after differentiation process (Figure 4b). After the first 5 days of culture, we analysed IDO expression by Western Blot and densitometry analysis. We observe that control tDC express 30% less IDO than iDCs (Figure 4b.a.b). Surprisingly, C15SEV‐iDC presents the highest IDO expression which is, respectively, 2.7 and 3.9‐fold more important than controls iDCs and tDC (Figure 4b.b). In parallel, we measured by HPLC the ratio of Kynurenine to Tryptophan and we observe that C15SEV‐iDC secrete significantly more Kynurenine than Tryptophan compared to control iDCs and tDC (approximatively 1.55‐fold more) (Figure 4b.c). In addition to IDO catabolizing tryptophan, it has been described that interleukin 4‐induced gene 1 (IL4I1), a secreted enzyme belonging to the L‐amino acid oxidase family, is able to promote the cancer immunosuppressive capacity of aryl‐hydrocarbon receptors (AHRs) more potently than IDO1, previously recognized as the major Trp catabolic enzymes (Sadik et al., 2020). We also analysed the secretion of the metabolic regulatory enzyme IL4I1 by monocytes and moDC (Figure 4b.d). Interestingly, C15SEV‐iDC secrete much more IL4I1 than monocytes and tDC after differentiation.

### 3.5 During maturation, NPC tumour SEVs affect properties of moDCs

**FIGURE 5:**
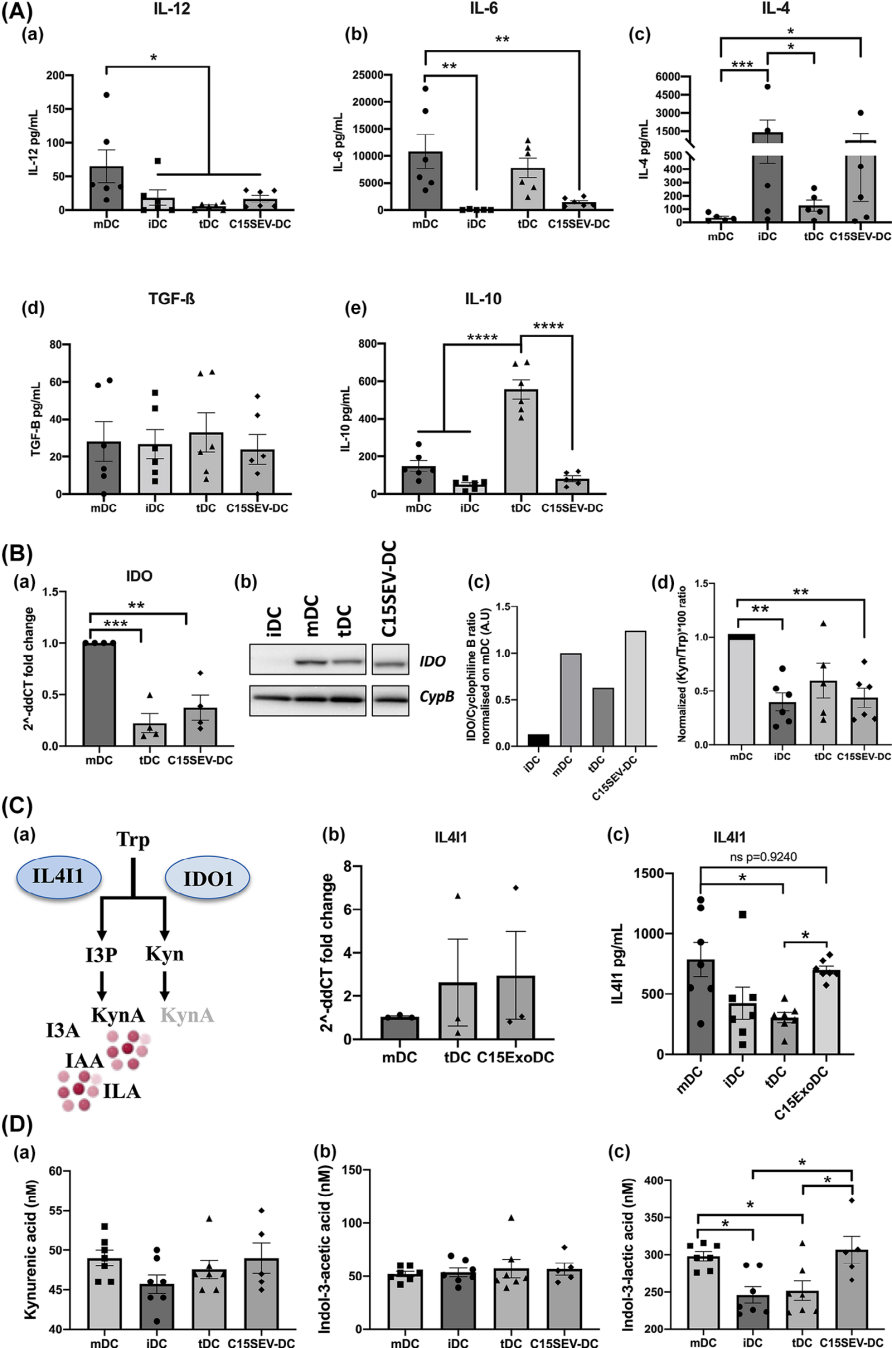
**Cytokine secretion and IDO—IL4I1 activity of moDCs after maturation. (A)** Dosage of cytokines secretion by ELISA in the culture supernatant of moDCs. **(a–c)** Secretion of the effector cytokines IL‐12, IL‐6 and IL‐4. **(d–e)** Secretion of immunoregulatory cytokines TGF‐ß and IL‐10. Results expressed in cytokines concentrations (pg/mL) from six independent experiments, MEAN ± SEM. Statistical differences between conditions were analysed by One‐Way ANOVA after outlier identification. *p < 0.05, **p < 0.01, ****p < 0.0001. **(B)** IDO expression and presence on moDCs and HPLC dosage of Tryptophan metabolites. **(B.a)** RT‐qPCR analysis of IDO expression in mDC, tDC and C15SEV‐DC. Data are representative of four and three independent experiments, respectively, normalised to gene expression in the mDC controls condition, mean ± SEM. **(B.b)** Testing of IDO expression by Western blotting were carried out. **(B.c)** The intensities of the IDO bands were quantified via ImageJ software and normalized to the Cyclophilin B (CypB) control and then normalized to the mDC control. **(B.d)** HPLC dosage of Tryptophan and Kynurenin. Results are express in (Kyn/Trp) ×100 ratio normalized on mDC**. (C)** Representation of the tryptophan (Trp) pathway **(C.a)**. Trp can be metabolised by IDO1 into kynurenine (Kyn), which will yield little kynurenic acid (KynA). In parallel, Trp is metabolised by IL4I1 to Indole‐3‐pyruvic acid (I3P). This oncometabolite, which is not very stable, is catabolised into 4 oncometabolites: kynurenic acid (KynA), indole‐3‐acetic acid (IAA) and indole lactic acid (ILA) and indole‐3‐aldehyde (I3A). **(C.b)** RT‐qPCR analysis of IL4I1 expression in mDC, tDC and C15SEV‐DC. Data are representative of three independent experiments, respectively, normalised to gene expression in the mDC controls condition, mean ± SEM. **(C.c)** ELISA dosage of IL4I1 enzyme in moDCs. Representative histogram of seven independent experiments, mean ± SEM. **(D)** HPLC determination of kynurenic acid **(D.a)**, indole‐3‐acetic acid **(D.b)** and indole‐3‐lactic acid **(D.c)**. Statistical differences between conditions were analysed by one‐way ANOVA or Kruskal Wallis after outlier identification. *p < 0.05, **p < 0.01, ***p < 0.001.

The same set of experiments were achieved after maturation (Figure 5). ELISA (Figure 5a.a‐e) studied effector cytokines IL‐12p70 and IL‐6 as well as immunomodulatory cytokines IL‐4, TGF‐β and IL‐10. After moDCs maturation, we measured high concentration of IL‐12p70 and IL‐6 by mDC (respectively, 94.16pg/mL ± 29 and 10803.2pg/mL ± 3165.3) and high secretion of IL‐6 by tDC (7805pg/mL ± 1782.7) (Figure 5a.a and 5a.b). However, C15SEV‐DC shows a significant decrease secretion of both IL‐12p70 and IL‐6, respectively, 5.57 and 7.47‐fold less compared to control mDC (Figure 5a.a and 5a.b). Concerning immunoregulatory cytokines, we investigated the production of IL‐4. Results demonstrated that C15SEV‐DC secrete significantly more IL‐4 compared to mDC and tDC control (Figure 5a.c). In addition to IL‐4, we measured TGF‐ß and IL‐10 production. However, C15SEV‐DC did not have difference in TGF‐ß and IL‐10 secretion compared to control mDC (Figure 5a.d and 5A.e). Only control tDC show, as expected, an important IL‐10 secretion compared to other DCs (Figure 5a.e).

Identically as for the differentiation process, we analysed IDO expression and activity after maturation process of all moDCs (Figure 5b.a‐d). However, after maturation, IDO mRNA is significantly less expressed in C15SEV‐DC compared to mDC (Figure 5b.a). Study of IDO by Western Blot and densitometry analysis showed a similar presence in C15SEV‐DC with the same protein level compared to controls mDC (Figure 5b.b.c.). HPLC dosage showed that IDO activity is twice lower for C15SEV‐DC and iDCs compared to controls mDC (Figure 5b.d). While the total protein seemed to be equal in between the mDC and C15SEV‐DC, both the upstream (transcriptomic activity) and downstream (enzymatic activity) activities of IDO pathway was downregulated in C15SEV‐DC at D7.

It is known that IL4I1 activates AHR via the generation of oncometabolites such as kynurenic acid (KynA), indole acetic acid (IAA) and indole lactic acid (ILA) from Trp (Figure 5c.a). Thus, we analysed the expression and production of IL4I1 (Figure 5c.b and 5C.c). We observed that IL4I1 is similarly expressed between mDC, tDC and C15SEV‐DC (Figure 5c.b). These results are confirmed by the enzyme dosage by ELISA showing no differences between C15SEV‐DCs and mDCs in term of IL4I1 production (Figure 5c.c). Finally, we measured the amount of kynurenic acid, indole acetic acid and indole lactic acid metabolites from the supernatants of the moDCs (iDCs, mDCs, tDCs and C15SEV‐DC) by HPLC (Figure 5d.a‐c). We could observe that the mDCs produced more kynurenic acid than the iDCs and more indole lactic acid than iDCs and tDCs (Figure 5d.a and D.c). In contrast, indole acetic acid is similarly present in the supernatants of moDCs. For C15SEV‐DC, we note that the production of the different metabolites is identical to that of mDCs (Figure 5d.a‐c).

### 3.6 Quantitative analysis shows a significant difference in the oxygen consumption rate for C15SEV‐DC

**FIGURE 6:**
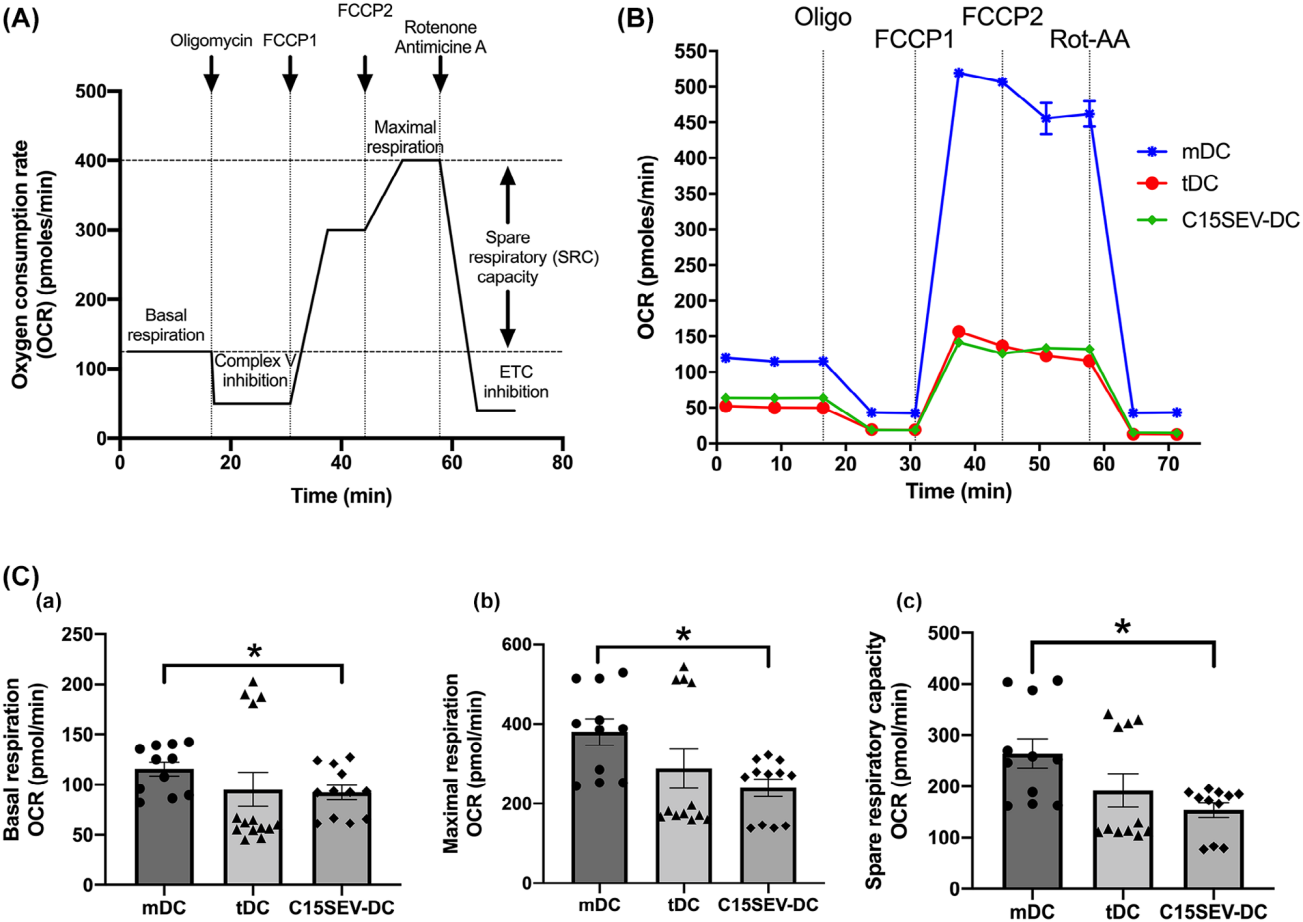
**Characterization of mitochondrial function of mDC, tDC and C15SEV‐DC. (A)** Schematic representation of a real‐time mitochondrial respiration. OCR analysis starting from basal respiration and after the addition of oligomycin (complex V inhibitor), FCCP (maximal respiration induction), and rotenone/antimycin A mixture (electron transport chain [ETC] inhibitors). The mitochondrial spare respiratory capacity (SRC) (maximal subtracted from basal respiration) is derived from the OCR curve. **(B)** Representative kinetic measurement of mitochondria OCR (pmoles/min) in mature (mDC, blue), tolerogenic (tDC, green) and co‐cultured with C15SEVs (C15SEV‐DC, dark green) moDCs by the addition of oligomycin (Olig), FCCP, and rotenone/antimycin A (Rot‐AA) over time. **(C)** OCR quantification of basal respiration **(a)**, maximal respiration **(b)** and SRC **(c)** of mDC, tDC and C15SEV‐DC, respectively. The data are representative of three independent experiments, MEAN ± SEM. Statistical differences were analysed by one‐way ANOVA after outlier identification, *p < 0.05.

Our data demonstrated that C15SEV‐DC have tolerogenic properties and are not capable to activate T cells properly. Maturation process require important metabolic adaptations to give rise to DC survival, migration, and the activation of specific immunity. To test if C15SEV‐DC have some modifications in their metabolism pattern we analysed oxidative phosphorylation (OXPHOS) of moDCs. To do this, we measured the oxygen consumption rate (OCR) of all moDCs (after maturation) in real time using Seahorse technology, as described in Figure 6A. We observed different levels of both basal and maximal respiration in between mDC, tDC and C15SEV‐DC (Figure 6B). Quantitative analysis shows that there is a significant difference in basal and maximal OCR between mDC and C15SEV‐DC (Figure 6C.a‐b). We further analysed SRC (Spare Respiratory Capacity) and C15SEV‐DC have a lower SRC than mDC (Figure 6C.c).

### 3.7 C15SEVs and CCL19 gradient significantly attract C15SEV‐DC

To assess the importance of NPC derived SEVs on the development of NPC tumour, we decided to analyse their ability to attract different moDCs. We further evaluated the migration comportment of mDC, tDC and C15SEV‐DC. DC were seeded into μ‐Slide chemotaxis^3D^ chambers and exposed to culture media, gradient of CCL19 (100 µg/mL) as a control or C15SEVs gradient (5 µg/mL) decreasing from the right to the left (Figure 7A). Supplementary data 1 display videos of mDC, tDC and C15SEV‐DCs migration under C15SEV gradient over time after displacement analysis using Imaris software. Whereas mDC and C15SEV‐DC have migratory capacity under control condition with culture media, tDC appear not to move and looked paralysed (Figure 7A.a‐c). When DCs were subjected to CCL19 gradient during all the experiment (toward the left along the X‐axis as compared to the Y‐axis, Figure 7A.d‐f) both mDC and C15SEV‐DC migrated toward this chemokine while tDC do not migrate. Concerning C15SEVs gradient (Figure 7A.g‐i), we observed that some mDC move toward C15SEVs gradient and the other seems to migrate in the opposite direction while tDC do not migrate. Interestingly, C15SEV‐DC migrate more importantly toward C15SEVs gradient (Figure 7A.i).

FIGURE 7
**In vitro migration of moDCs toward culture media, CCL19 or C15SEVs gradient. (A)** Migratory comportment of mDC (left panels), tDC (middle panels) and C15SEV‐DC (right panels) monitored by time lapse imaging for 6 h while subjected culture media **(a‐c)** or to a right to left (x axis) decreasing gradient of CCL19 **(d‐f)** and C15SEVs **(g‐i)** (indicated by the arrows on the top graphs). Trajectories of different DCs that migrate toward CCL19 or C15SEVs gradient are in green while the noes that moved against the gradient are in red. The black diamond symbol on graphs indicates the centre of mass (COM) of the cell population at the end of the experiment. **(B) Quantitative analysis of the migratory comportment illustrated in** Figures 7A **.a‐i**. Quantitative analysis of moDCs migration including **(a)** velocity, **(b)** parallel and perpendicular centre of mass (COM)**, (c and d)** parallel and perpendicular forward migration index (FMI), **(e)** directness and **(f)** p‐value of the Rayleigh test of mDC, tDC and C15SEV‐DC. Graphs **(a, c, d and e)** are expressed in MEAN ± SEM from two independent experiments for culture media (mDC and tDC), CCL19 gradient, C15SEVs gradient for mDC migration and one experiment for tDC under C15SEVs gradient and C15SEV‐DC under culture media and C15SEVs gradient. Statistical differences between conditions were analysed by kruskal Wallis test with *p < 0.05 considered as significant, **p < 0.01, ***p < 0.001, ****p < 0.0001.

**Figure:**
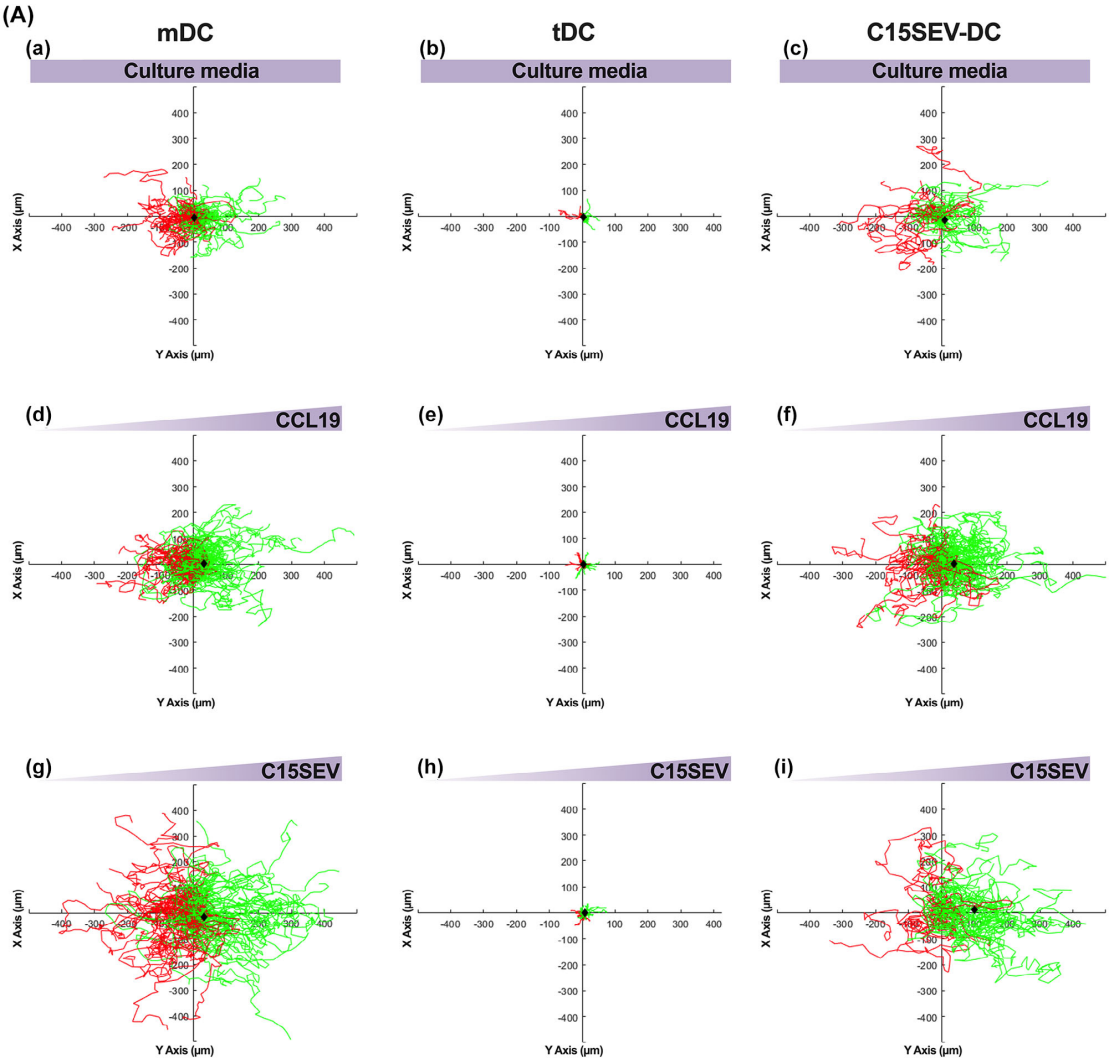

**Figure:**
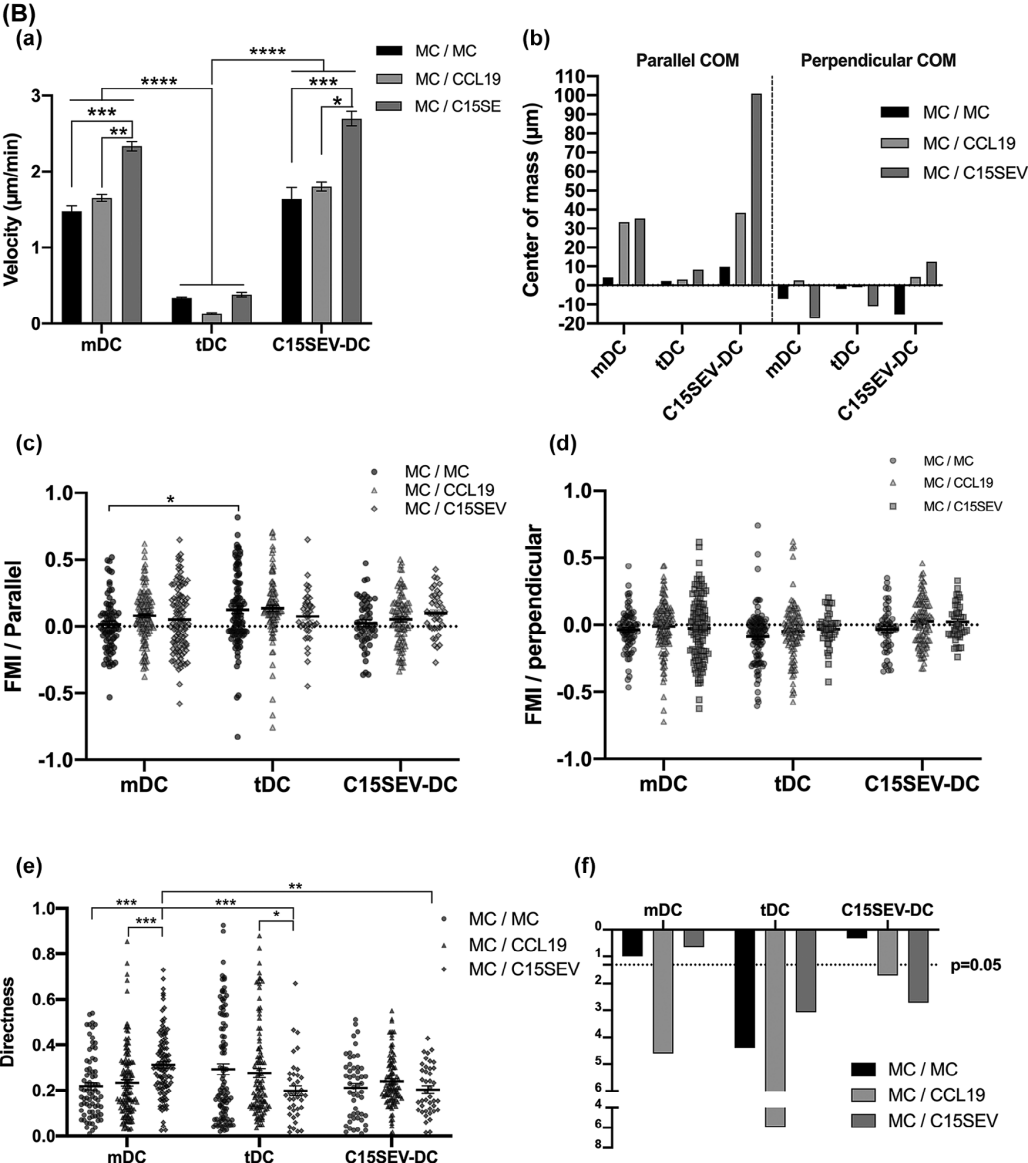

Quantitative analysis of the migration was then performed by measuring parameters related to speed, directionality (velocity, parallel and perpendicular FMI, directness, *p*‐value of the Rayleigh test) of mDCs, tDCs and C15SEV‐DCs and the average displacement of their centre of mass (COM) parallel and perpendicular to the CCL19 and C15SEVs gradient (Figure 7B). Velocity of DCs was weakly affected in the presence of CCL19 compared to velocity in control culture media condition. While C15SEVs gradient increase significantly migration speed of both mDCs and C15SEV‐DCs (Figure 7B.a). Despite the unchanged velocity in CCL19 gradient, CCL19 strongly attracted mDCs as indicated by parallel COM displacement (Figure 7B.b and black diamond symbols on migration plots in Figure 9A.d and 9A.f) and by the increase in positive parallel FMI value (Figure 7B.c) followed by an almost null perpendicular FMI (Figure 7B.d) relative to the gradient. In addition to increased migration speed, C15SEVs seems to attract mDCs in the same way that CCL19 visible by increase parallel COM (Figure 7B.b and black diamond symbols on migration plots in Figure 9A.g) but parallel FMI is not significant as for CCL19 gradient (Figure 7B.c). For C15SEV‐DCs, we observe as for mDCs an attraction by CCL19 as indicated by parallel COM (Figure 7B.b) and by almost null perpendicular FMI (Figure 7B.d). Interestingly, we observe that C15SEVs attracted even more strongly C15SEV‐DCs with an important parallel COM displacement (Figure 7B.b and black diamond symbols on migration plots in Figure 7A.i) accompanied by significant positive parallel FMI and null perpendicular FMI (Figure 7B.c and 7B.d). Furthermore, directness (Figure 7B.e) and Rayleigh test (Figure 7B.f), respectively, indicate, that mDCs migration followed more direct trajectories in the presence of an C15SEVs gradient than CCL19 and that mDCs are significantly guided toward CCL19 compared to C15SEVs gradient (significant Rayleigh test with CCL19 gradient Figure 7B.f). For C15SEV‐DC, directness and Rayleigh test, respectively, indicate that under CCL19 and C15SEVs gradient, these cells do not have straightforward trajectories and that C15SEV‐DC are significantly both attract toward CCL19 and C15SEVs gradient (Figure 7B.e and 7B.f). Finally, in accordance with Figure 7A, tDC have a low migration capacity resulting in a migration speed and COM in CCL19 and C15SEVs gradient close to zero. With this lack of migration, other results such as the Rayleigh test are not interpretable.

### 3.8 Galectin‐9S affect DC function and a neutralizing anti‐Gal9 antibody could reverse this effect

**FIGURE 8:**
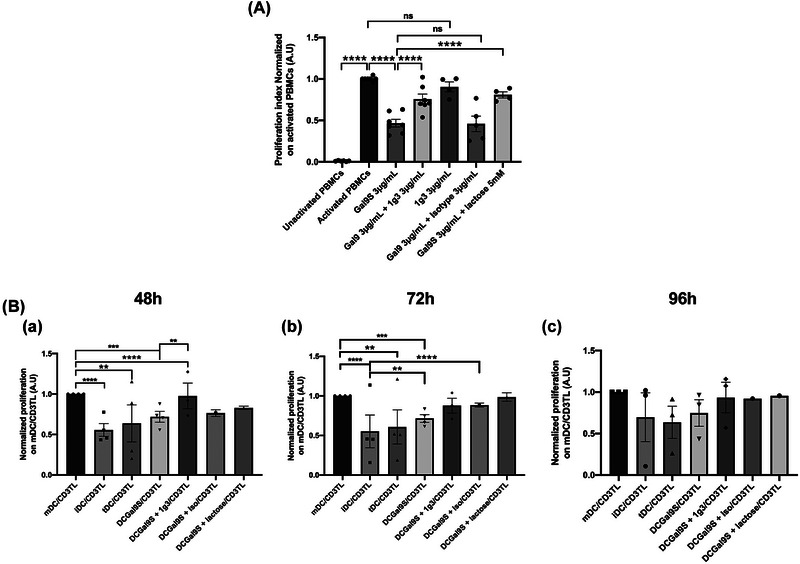
Immunosuppressive properties of recombinant Galectin‐9S and suppressive function of DCGal9S reverse by the use of an antibody neutralizing galectin‐9. (A) Proliferation assay of PBMCs co‐culture with recombinant galectin‐9S (3 µg/mL) ± an antibody neutralizing galectin‐9 (1g3) (3 µg/mL) or isotype IgG1K (3 µg/mL) or lactose (5 mM) and with anti‐gal9 antibody alone (1g3) after 48 h of culture. Results are expressed in proliferation index normalized on activated PBMCs, MEAN ± SEM. Results are representatives of three independents experiments for the condition 1g3 and Gal9S + lactose, four independent experiments for the condition Gal9S + Iso and seven independents’ experiments for the conditions Gal9S and Gal9S + 1g3. Statistical differences between activated PBMCs and other conditions were analysed by one‐way ANOVA with ****p < 0.0001 consider to be significant. (B) Suppression assay of CD3+ T cells co‐cultured for 48 h (B.a), 72 h (B.b) or 96 h (B.c) with DCs that were pre‐cultured with Galectin‐9S (3 µg/mL) ± an antibody neutralizing galectin‐9 (1g3) (3 µg/mL) or IgG1K isotype (3 µg/mL) or lactose (5 mM). Results are expressed in proliferation index normalized on mDC/CD3TL proliferation, MEAN ± SEM, *n* = 4 (iDC, mDC, tDC et DCGal9S), *n* = 3 (DCGal9S + 1g3) at 48 and 72 h. *n* = 3 (iDC, mDC, tDC et DCGal9S, DCGal9S + 1g3) at 96 h. *n* = 1 for the others conditions. Statistical differences between conditions were analysed by one‐way ANOVA or kruskal Wallis after outlier identification with **p < 0.01, ***p < 0.001, ****p < 0.0001 consider to be significant.

In order to understand by which mechanism the immunosuppressive properties of NPC SEVs on DC could be explained, we focused our attention on Galectin‐9, a highly immunosuppressive protein carried by NPC SEVs (Figure 1C). We first decided to evaluate the impact of recombinant Galectin‐9 on the immune system in order to confirm the efficacy of a neutralizing antibody targeting Galectin‐9 patented by the laboratory (1g3 clone). For this, we cultivated human PBMCs with recombinant galectin‐9 S isoform (Gal9S, 3 µg/mL) blocked or not with the neutralizing antibody (3 µg/mL) or an isotypic control (3 µg/mL) or with lactose (5 mM) as a natural inhibitor. We proceeded a proliferation assay after 48 h of culture (Figure 8A). Firstly, we confirmed the immunosuppressive properties of the Gal9S with a decrease of 54% of the proliferative capacity of PBMCs in comparison to activated ones. Secondly, we confirmed the efficacy of the neutralizing antibody 1g3 with a restoration of 29% of PBMCs’ proliferation. We validated no impact of the antibody anti‐Gal9 alone, no impact of the isotypic control and an inhibition of Gal9S suppressive effect by the lactose (Figure 8A).

To assess if Galectin‐9 could have an impact on human dendritic cells maturation, we generated moDC treated with recombinant Galectin‐9S (DCGal9S). As previous assessed with C15SEVs, we evaluate the functional properties of moDC after 7 days of culture by measuring CD3+ T cells proliferation after a co‐culture between moDCs and total heterologous CD3+ T cells (Figure 8B). DCGal9S significantly decrease CD3+ T cells proliferation after 48 h (29%) and 72 h (28%) of culture (Figure 8B.a‐b). In a second step, we generated moDC treated with recombinant Galectin‐9S (DCGal9S) blocked or not with the neutralizing antibody (DCGal9S + 1g3) or an isotypic control (DCGal9S + Iso) or lactose (DCGal9S + lactose). In very interesting way, blocking Gal9S with the neutralizing antibody reverse the immunosuppressive effect of DCGal9S by restoring CD3+ T cells proliferation at 48 h (26%) (Figure 8B.a).

These results demonstrated for the first time that galectin‐9S shifts the activation function of DCs to a regulatory function, which can be secondarily inhibited by the addition of the 1g3 blocking antibody.

### 3.9 Recombinant galectin‐9S induce DC with mature properties

**FIGURE 9:**
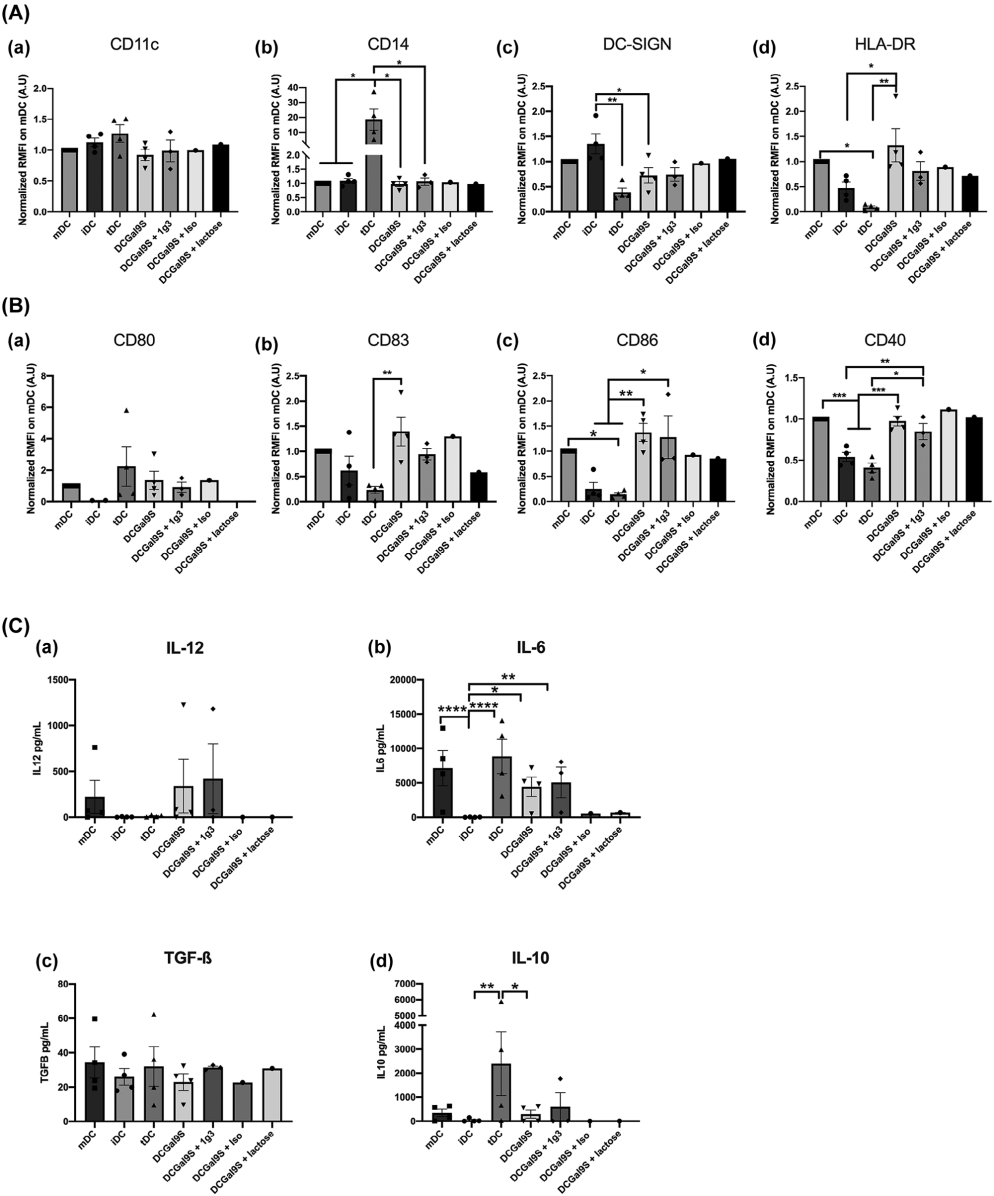
**Characterization of moDCs pre‐cultured with recombinant Gal9S after maturation. (A)** Expression level of surface markers CD11c, CD14, DC‐SIGN, HLA‐DR **(A.a‐d)**, CD40, CD80, CD83 and CD86 **(B.a‐d)** in mature (mDC) immature (iDC), tolerogenic (tDC) and co‐cultured with Gal9S (DCGal9S) blocked or not with 1g3 (DCGal9S + 1g3), isotype IgG1K (DCGal9S + Iso), lactose (DCGal9S + lactose) moDCs after maturation. The histograms are representative of four independent experiments for conditions iDC (except for CD80 where *n* = 2), mDC, tDC and DCGal9S, three independent experiments for DCGal9S + 1g3 and one experiment for DCGal9S + Iso and DCGal9S + lactose. The histograms are expressed as the median fluorescence ratio normalized to mDC; MEAN ± SEM. Statistical differences were analysed by one‐way ANOVA or Kruskall Wallis after outlier identification. *p < 0.05, **p < 0.01, ***p < 0.001, ****p < 0.0001. **(C)** Cytokine secretion of moDCs after maturation. Dosage of cytokines secretion by ELISA in the culture supernatant of moDCs. **(C.a‐b)** Secretion of the effector cytokines IL‐12 and IL‐6 **(C.c‐d)** Secretion of immunoregulatory cytokines TGF‐ß and IL‐10. Results expressed in cytokines concentrations (pg/mL) from four independent experiments for conditions iDC, mDC, tDC and DCGal9S, three independent experiments for DCGal9S + 1g3 and 1 experiment for DCGal9S + Iso and DCGal9S + lactose, MEAN ± SEM. Statistical differences between conditions were analysed by one‐way ANOVA or Kruskall Wallis after outlier identification. *p < 0.05, **p < 0.01, ***p < 0.001, ****p < 0.0001.

We, therefore, continued with the phenotypic analysis of DCGal9S, in particular the characterization of maturation membrane marker by flow cytometry (Figure 9A.B.). We observed that recombinant Gal9S does not affect the expression of maturation membrane markers in comparison to the maturation control mDC (Figure 9A.B.) and promotes the induction of phenotypically mature DC as seen for C15SEVs. Blocking Gal9S with 1g3 did not affect the expression of surface markers either.

The impact of Gal9S on DC maturation was then assessed by an evaluation of effector cytokines (IL‐12p70 and IL‐6) and immunomodulatory cytokine secretion (TGF‐β and IL‐10) by ELISA (Figure 9C). After moDCs maturation, we noticed no modification of DCGal9S secretoma with a secretion of effector cytokine (Figure 9 C.a‐ b) and immunoregulatory cytokines (Figure 9 C.c‐d) similar to mature control and therefore in favour of the activation of an immune response. Blocking Gal9S with 1g3 neither impacted the secretoma.

### 3.10 Blocking SEVs galectin‐9 partially reverse the immunosuppressive properties of NPC SEVs

**FIGURE 10:**
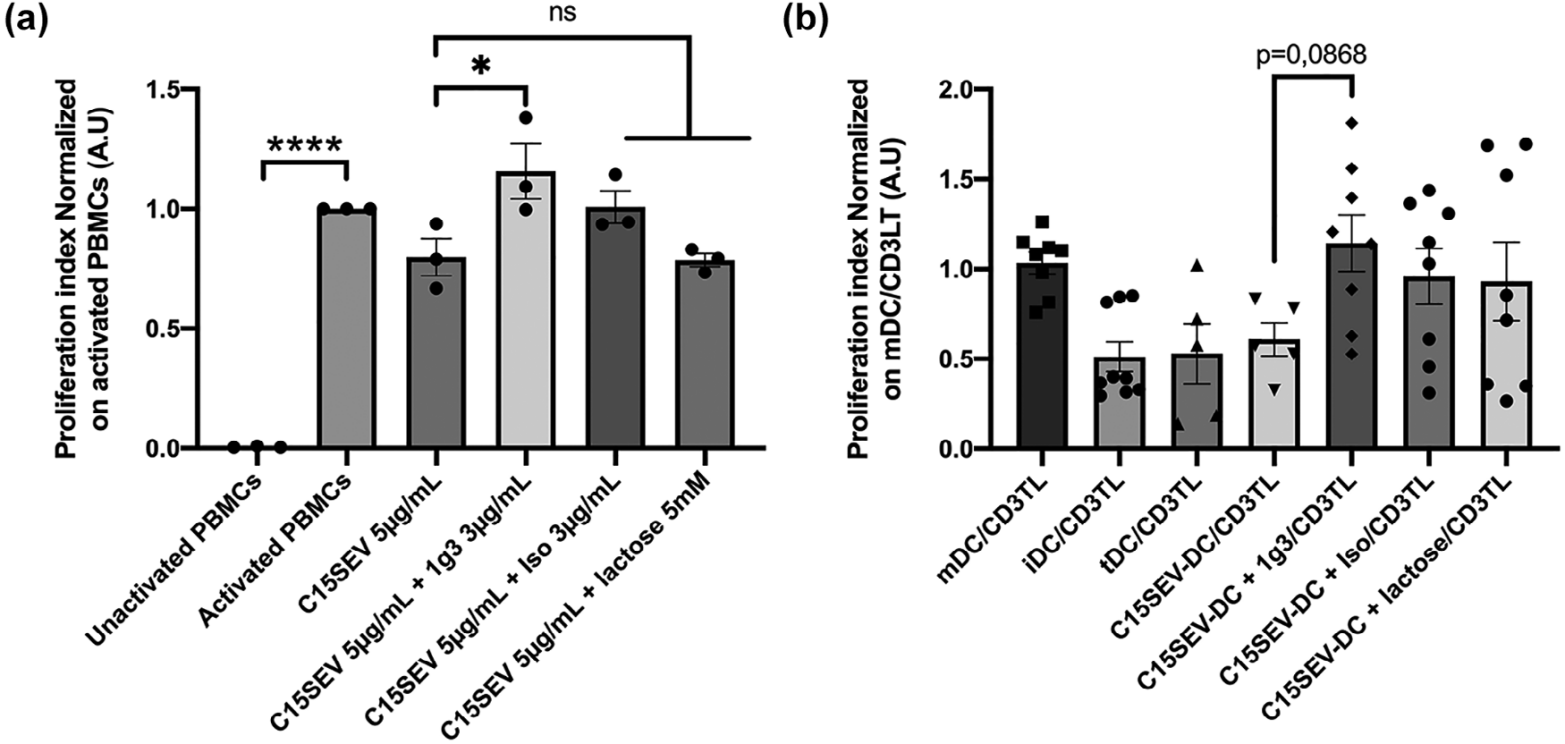
**Impact of blocking SEVs Galectin‐9 on their suppressive function (a)** Proliferation assay of PBMCs co‐culture with C15SEVs (5 µg/mL) ± an antibody neutralizing galectin‐9 (1g3) (3 µg/mL) or isotype IgG1K (3 µg/mL) or lactose (5 mM) after 72 h of culture. Results are expressed in proliferation index normalized on activated PBMCs, MEAN ± SEM. Results are representatives of three independents experiments. Statistical differences between conditions were analysed by one‐way ANOVA, **p < 0.01, ****p < 0.0001. **(b)** Suppression assay of CD3+ T cells co‐cultured for 96 h with DCs that were pre‐cultured with C15SEVs (5 µg/mL) ± an antibody neutralizing galectin‐9 (1g3) (3 µg/mL) or isotype IgG1K (3 µg/mL) or lactose (5 mM). Results are expressed in proliferation index normalized on mDC/CD3TL proliferation on three independents experiments, MEAN ± SEM. Statistical differences between conditions were analysed by one‐way ANOVA after outlier identification.

We first highlighted the fact that recombinant galectin‐9 has the ability to modify dendritic cells maturation in favour of a mature profile with tolerogenic properties. Secondly, we were able to show that this immunosuppressive property could be reversed by the addition of a blocking antibody. In this context, we investigated the importance of the immunosuppressive properties of galectin‐9 carried by NPC SEVs. For that purpose, we cultivated human PBMCs with C15SEVs (5 µg/mL) blocked or not with the 1g3 neutralizing antibody (3 µg/mL) or the isotypic control (3 µg/mL) or lactose (5 mM) as a natural inhibitor of Gal9. We proceeded a proliferation assay after 72 h of culture (Figure 10A). Interestingly, we observed a 29% significant restoration of PBMCs proliferation when we blocked SEVs'galectin‐9 with 1g3 in comparison to C15SEVs condition. More of that, as expected we noticed no significant effect of isotypic control and there was no restoration of the proliferation when using lactose. These results confirm the involvement of galectin‐9 in the suppressive effect of NPCSEVs.

As previously showed, moDC treated with NPCSEVs decrease CD3+ TL proliferative capacity testifying a tolerogenic function. In order to visualize and highlight the role of galectin‐9 in this mechanism, we finally performed a direct blockade of galectin‐9 using 1g3 on NPCSEVs. In that purpose, we perform a co‐culture between total heterologous CD3+ T cells and moDCs treated with C15SEVs (C15SEV‐DC) blocked or not with 1g3 (C15SEV‐DC + 1g3) or its isotypic control (C15SEV‐DC + Iso) or lactose (C15SEV‐DC + lactose) during 2 h at least (Figure 10B).

In a very interesting way, and for the first time, we showed that blocking the galectin‐9 carried by SEVs induce a partial recovery of CD3+ T cell proliferation (+46%; p = 0.0868), which declined at 96 h (42%) (Figure 10B) suggesting a possible Galectin‐9 mediated effect of NPC‐SEVs.

## 4 DISCUSSION

In order to understand why NPC escape from immunosurveillance the possible release of extracellular vesicles carrying immunosuppressives proteins from NPC cells was investigated (Keryer‐Bibens et al., 2006). With their proof of concept and previous further studies, Pierre Busson's team was able to confirm that NPC cells could release HLA class‐II positive SEVs containing Galectin‐9 conferring them immunosuppressive properties (Klibi et al., 2009). Important key points were assessed in their last studies since the authors were able to report those Gal9+ NPC derived SEVs to be selectively detected in plasma samples of NPC patients and mice xenograft with NPC tumours. Moreover, we and others have demonstrated that thanks to the protective role of EVs carriage Gal9 retains its ability to bind to T cell immunoglobulin and mucin domain‐containing protein 3 (TIM3) triggering apoptosis of specific Th1 cells (Lhuillier et al., 2015; Zhu et al., 2005). Furthermore, in the continuing effort to decipher the role of these nanovesicles, we proved that their interaction with Tregs, resulted in an increase of their suppressive properties and function. These NPC‐derived SEVs also induced a shift of the conventional T cell lymphocytes into Tregs (Mrizak et al., 2015).

In this present work, we demonstrated for the first time that the shaping of conventional DCs by the same NPC‐derived SEVs induces impaired DCs. It is now clear that DCs populations are heterogenous in term of ontogeny as well as function. Indeed, while fully mature DCs favour T cell stimulation, tolerogenic DCs (tDC) regulate the immune response. tDC can be characterized by a semi‐mature phenotype and an ability to block effector T cell response while promoting Treg function and differentiation. The semi‐mature tDC phenotype is notably characterized by lower expression levels of some co‐stimulatory markers (Patente et al., 2018). In very recent years, new sequencing technologies, including single‐cell RNA sequencing, have made possible to identify across different DCs lineages a common DC program named “mature DCs enriched in immunoregulatory molecules” or mature regulatory DCs (mregDCs) (Kvedaraite & Ginhoux, 2022; Maier et al., 2020; Zilionis et al., 2019). MregDCs are characterized by a signature specific to DC maturation (CD40, CD83 or CD86), migration (CCR7 and ICAM), immunosuppression molecules (PD‐L1, PD‐L2 and CD200) (Maier et al., 2020). The last authors described the existence of the mregDCs program by canonical conventional type 1 and 2 dendritic cells (DC1s and DC2s) that have a function in immune regulation to avoid overactivation of immune cells. As expected, in tumour context, mregDCs have pro‐tumoural function and can present a lack of IL‐12 expression and production. In the present work, we observed that C15SEV‐iDC showed an immature phenotype and a lack of LT activation function similar to classical iDC, but with stronger immunoregulatory markers. In addition, C15SEV‐iDC are resistant to maturation, giving rise to C15SEV‐DC that not only block T cell activation, but also exhibit a mature phenotype with expression of presentation markers (HLA‐DR) and costimulation markers (CD40, CD80, CD83, CD86) in accordance with the preview's description of mregDCs (Cheng et al., 2021; Maier et al., 2020). We analysed oxidative phosphorylation metabolism in DCs by measuring oxygen consumption over time. Analysis indicates a reduced ability of these C15SEV‐DCs to use oxidative metabolism as an energy source with lower basal, maximal OCR and lower SRC. In line with our results, Santos and colleagues previously described that in the context of hepatocellular cancer (HCC), DCs exposed to α‐fetoprotein exhibit a reduction in fatty acid metabolism and mitochondria metabolism with basal oxygen consumption rate reduction (Santos et al., 2019). These modifications explain in part a mechanism responsible for immune suppression in HCC. Further analyses are needed in the context of NPC to understand in depth the possible metabolic changes of DCs in the presence of NPC derived EVs. In fact, fatty acid metabolism is notably well described as a pathway used by regulatory cells including regulatory DCs to achieve their regulatory function and induce immune suppression (Pearce & Everts, 2015; Zeyda et al., 2005).

In our work, we also observed during differentiation state, that DCs, in the presence of NPC derived EVs (C15SEV‐iDC), produce higher levels of IL‐10 and exhibit a stronger IDO activity and like immature DC are not able to activate T lymphocytes. These results are consistent with other studies showing, in particular that EVs derived from mesenchymal stem cells induce regulatory DCs that impaired T cell activation and produce high amounts of IL‐10 and IL‐6 (Enrica et al., 2016). In addition, Valenti et al. have shown that exosomes derived from melanoma patients and cell lines modify the differentiation of monocytes, generating myeloid suppressor cells. These cells express very little CD80/CD86 protein and produce abundant IL‐6, TNF‐α and TGF‐ß (Valenti et al., 2006). In other cases, it has been observed that tumour derived EVs can enhance tumour progression by inducing MDSC, promoting immunosuppression (Wang et al., 2015). Concerning our results, this early IL‐10 production and IDO activity could be related to the presence of the EBV‐LMP1 on C15SEVs. In fact, it was described that in its first transmembrane domain, LMP1 have an immunosuppressive conserved sequence LALLFWL responsible for IL‐10 production by cells (Dukers et al., 2000). Identically, Tsai et al. described that LMP1 expressed in B cells can inhibit humoral response and enhance IDO1 expression and activity by the activation of NF‐kB pathway (Chaves et al., 2001; Tsai et al., 2018; Tu et al., 2017). Additionally, it was shown that metabolites of tryptophan produce by IDO can convert immunogenic DC into tolerogenic ones (Belladonna et al., 2006; DeVito et al., 2019). In fact, IFN‐γ activate IDO on DCs, resulting in a specific tolerance through the induction of tryptophan starvation and the production of kynurenines. In return, this IDO activated DCs secrets Kynurenine to activate regulatory response of other DCs in which IDO enzyme is not active. Then those DCs induced under low tryptophan, and high metabolite conditions can lead to deactivation of T cells and thus to suppression of the anti‐tumour immune response (Von Bubnoff et al., 2011). Gargaro et al. also demonstrate that IDO expressing cDC1 cells indirectly induce immunoregulatory capacities of the cDC2 subpopulation thanks to their production of kynurenine (Gargaro et al., 2022). In our hands, mature DC expressed the higher level of IDO after the maturation phase, with a complete activation process from upstream gene expression to downstream enzymatic activity. However, this pro‐regulatory capacity did not seem to be sufficient to overcome T cell stimulation, probably due to the counteraction of all the costimulatory molecules they contain and the absence of inhibitory signals. This feature has been well described by others who have discussed the ambivalent and paradoxical relevance of IDO expression by mature DC, particularly in cancer therapy (Harden & Egilmez, 2012; Krause et al., 2007). On the contrary, NPCSEV‐DCs showed impaired production of proinflammatory cytokines IL‐6 and IL‐12. In addition, to the lack of IL‐12 production, IDO is neither express nor active in these DCs visible by RT‐qPCR analysis (Figure 5 and Supplementary data 6), showing a significant decrease in IDO expression and Trp metabolites production. A critical link in between IL‐4 and IDO has been described. Indeed, IL‐4 by its ability to counteract IFN‐γ signalling can potently reduce IFN‐γ induced IDO production. This feature being usually accompanied with a decrease of IL‐1, IL‐6, and TNF‐α inflammatory cytokines secretion (Musso et al., 1994). In this study, authors were able to prove that IDO is even directly downregulated via IL‐4 signalling itself, indicating an immunologic control of IDO. Beyond the transcriptional description of mregDCs, Maier et al. highlight the negative role of the Interleukin‐4 (IL‐4) that they secrete. Indeed, IL‐4 plays a pivotal role in the detriment of the effector function of mregDCs, in particular via the antagonistic blocking of IFN‐γ secretion, which is itself necessary for the production of IL‐12. The relation between tumour growth and IL‐4 has been reported for numerous types of cancer expressing IL‐4 receptor (IL‐4R) including NPC. In fact, Budiani and colleagues reported an increase in IL‐4 production both at the tumour level and in the sera of NPC patients compared to controls (Budiani et al., 2011; Shurin et al., 1999). IL‐4 plays a critical role in tumour development, being associated with aggressiveness and metastasis potential (Kobayashi et al., 1998), and most recent studies indicate that endogenous IL‐4 can favour resistance to apoptosis via IL4‐R signalling (Li et al., 2008). Very interestingly, we also pointed out, the ability of NPCSEV‐DCs to secrete much more IL‐4 than other classical DCs, either mature or tolerogenic. Therefore, altogether, our results clearly indicates that the emerging NPCSEV‐DCs belong to the newly described mregDCs subtype.

More recently, in an attempt to understand the failure of phase III clinical trial targeting IDO associated to immune checkpoint blockade, Sadik et al. has described, and largely proved, that interleukin 4 (IL‐4)‐induced gene 1 (IL4I1), a secreted enzyme belonging to the L‐amino‐acid oxidase family, was able to promote Aryl‐Hydrocarbon Receptor (AHR)‐driven cancer immunosuppressive ability. This potency was even described as more effective than for IDO1 or IDO2, hitherto recognized as the main Trp‐catabolic enzymes in the TME (Boulland et al., 2007; Sadik et al., 2020). Interestingly NPCSEV‐DCs produce IL4I1 and the fact that mDCs produce as much as IL4I1 than tolerogenic DCs, but without suppressive influence, could probably be explained by a restriction of its activity (or the one of its metabolites) in the effector microenvironment, while emphasized in an immunosuppressive one. A point that need to be clarified with further experiment. Thanks to our results, we can assume that the immunosuppressive properties do not only require or rely an increase in IL4I1 secretion, but probably much more via the concomitant action of the immunosuppressive molecules released after NPC derived EVs contact. Nevertheless, it is interesting to note that according to the TCGA dataset covering more than 500 H&N cancer patients, IL4I1 is significantly more than two‐fold overexpressed in tumour tissue than in normal ones (Supplementary data 7A). While looking at the most probable source of the enzyme into the TME of H&N cancer patients, one can observe a strong correlation with numbers of immune infiltrating cells, and among them myeloid cells are the most correlated (Supplementary data 7B); confirming our data in NPCDC *ex vivo* assays. Furthermore, when examining the impact of IL4I1 expression in the survival of H&N cancer patients, we found that, although it does not appear to impact on the overall survival (OS) of patients, there is a significant difference in relapse‐free survival (RFS, Supplementary data 7 C.a and C.b). As a result, the high IL4I1 expression is, the more likely patients are to relapse, confirming that IL4I1 could be a good candidate for therapeutic development. It is important to note that the main immune checkpoints described to date (PD1, PDL1, CTLA4) have exactly the same profiles for most cancers. Overall, our data are in agreement with several other recent publications (Molinier‐Frenkel et al., 2019; Sadik et al., 2020), and it can be assumed that IL4I1 is a novel immune checkpoint, and an interesting target for the development of new antitumor therapies. Finally, Maier et al., in their last description of mregDCs indicates that, IL4I1 was upregulated among other Th2 markers. Altogether, this information supports that IL‐4 upregulation and consequently IL4I1 secretion represent a real hallmark of mregDCs, explaining their detrimental role in cancer progression and resistance (Maier et al., 2020). The last authors suggested that some help in counteracting tumour resistance to treatment in lung cancer patient could come from an IL‐4 blockade, and according to our results this adjuvant treatment could surely be of great help to overcome resistance and delay recurrence in non‐responding NPC patients.

In NPC context, another particularly interesting candidate is Galectin‐9 (Gal9) that is found on NPC SEVs (Keryer‐Bibens et al., 2006; Pioche‐Durieu et al., 2005) and has various immunosuppressive capacities that greatly impact on the anti‐tumour response. Indeed, galectin‐9 lead to Th1 cells apoptosis (Zhu et al., 2005), decrease secretion of pro‐inflammatory cytokines (IL‐17, IL‐12 and IFN‐γ (Seki et al., 2008), and favor MDSCs or iTreg generation (Seki et al., 2008; Wu et al., 2014; Zhang et al., 2020). More importantly, it was reported that exosomes isolated from the cerebrospinal fluid (CSF) of patients with glioblastoma multiform (GBM) contain galectin‐9 that can interact with the TIM3 receptor on DCs. This interaction inhibits antigen recognition, processing and presentation by tumour‐infiltrating DCs, resulting in the failure of the cytotoxic T‐cell‐mediated anti‐tumour immune responses (Ming et al., 2020). Studies have demonstrated the importance of the Galectin‐9/TIM3 interaction in the regulation of dendritic cell function in cancer. Blocking TIM3 receptor on cDC1 in breast cancer has shown an improved anti‐tumour response after chemotherapy by promoting intra‐tumour CD8+ T response mediated by CXCL9 expression. Blocking Galectin‐9 in this same model showed the same results as anti‐TIM3 with an increase of CXCL9 expression confirming the interaction between these two actors in DC modulation effect (de Mingo Pulido et al., 2018). Recently, it has been described in the context of chronic coxsackievirus B3 infection that Gal9 promotes LT CD4 anergy, enhances Treg emergence and IL‐10 and IL‐4 production (Lv et al., 2011). This result, in line with ours, led us hypothesize exosomale Gal9 could be an interesting candidate to explain our results especially in the context where we had shown that NPC derived SEVs directly influenced Tregs (Mrizak et al., 2015).

TIM3, the main receptor of Gal9, is a transmembrane protein member of the TIM family of immunoregulatory receptors with immunosuppressive functions (Wolf et al., 2020). There are no classical inhibitory signalling motifs in the cytoplasmic tail of TIM3 which contains only five tyrosine conserved between mice and humans. Among those tyrosines, Tyr256 and Tyr263 allow interactions with HLA‐ B‐associated transcript 3 (BAT3) (Rangachari et al., 2012) and the tyrosine kinase FYN (Lee et al., 2011). According to current knowledge, when TIM3 is free of ligand, BAT3 is retain at its cytoplasmic tail and helps in activating signalling of T cell (Clayton et al., 2014). However, upon Gal9 binding, TIM3 oligomerize and both Tyr256 and Tyr263 are phosphorylated, which secondly triggers BAT3 release and unleash immunosuppressive function of TIM3 (van de Weyer et al., 2006; C. Zhu et al., 2005). Although firstly described as a T cell membrane molecule (Monney et al., 2002), Tim3 was found to be expressed in several myeloid cells such as neutrophils (Anderson et al., 2007; Huang et al., 2020), monocytes/macrophages (Nakayama et al., 2009; Vega‐Carrascal et al., 2014; Zhang et al., 2020) and more recently on Dendritic cells (Chiba et al., 2012; de Mingo Pulido et al., 2018), usually at higher levels than observed in T cells. The activation of the Gal9/Tim3 axis on myeloid cells regulate their polarization and turn them into immunoregulatory cells. For example, it has clearly been demonstrated that Gal9 could promote M2 type macrophages by deeply modifying expression patterns of transcription factors with an increase of STAT3 phosphorylation as well as cytokines release with a IL‐12/IL‐23 axis blockade combine to an increase of IL‐10 and TGF‐β secretion (R. Lv et al., 2017; Ma et al., 2013). On intratumor CD103+ cDC1s, TIM‐3 decrease the expression of Cxcl9 and Cxcl10 chemokines, implicating its role in effector T cell fate (de Mingo Pulido et al., 2018). More recently, the same team has described a mechanism by which TIM‐3 suppresses DNA sensing through the cGAS‐STING pathway through the binding of Gal9 (de Mingo Pulido et al., 2021). This idea was recently further reinforced by the demonstration of the direct role played by Gal9 on the degradation of STING shifting the myeloid compartment through the immunoregulatory part promoting immunosuppressive microenvironment in Gal9 secreting tumour (Zhang et al., 2020). The latter's research were also perfectly correlated with another recent one indicating that a blockade of TIM3 could not only increase antitumor CD8 T cells activation and IFN‐γ secretion, but would also favour cDC1 cells and CD8 T cells to colocalize within the tumour, amplifying the chance to mount an appropriate antitumor response both in time and space (Gardner et al., 2022). Moreover, it has been very recently described that BAT3 is a specific and endogenous regulator of tolerogenic DCs phenotype and function able to favour a pro‐tumour T cell compartment (reduction of TH1 cells, and increase in Treg cells) (Tang et al., 2022). Finally, in term of metabolic activity, it is now clear that TIM3 and Gal9 act as enhancer of immunoregulatory function through a shift in glycolysis at the expense of OXPHOS. This has been well described for Treg cells (Banerjee et al., 2021) and due to well established knowledge about tolerogenic DCs, one can speculate the same feature for them (Dáňová et al., 2015). Altogether, these studies indicate clearly the major role displayed by the Gal9/TIM3 axis, both in CIS and TRANS, in the emergence of regulatory DCs, notably within the tumour microenvironment. Indeed, one can resume that Gal9 binding to TIM3 on DC reinforce its membrane stability, allows TIM3 to oligomerize in order to deliver its immunoregulatory signal. This signal relies both on the degradation of STING and on the release of BAT3 protective adapter from the cytoplasmic tail of TIM3. These two complementary signals lead to the activation of STA3 and NFkB transcription factors resulting in blocking IL‐12 secretion in favour of IL‐10 and IL‐6 release (Barton, 2006), such as described for tumour cells (Zhang et al., 2018). Because of this dysregulation, Dendritic Cells lose their ability to activate antitumor T cells response and rather facilitate tolerance in T cells via multiple mechanisms already described elsewhere such as T cell anergy, T cell depletion or conversion into FOXP3 regulatory T cells (Iberg et al., 2017).

In the present study, we described the emergence of tolerogenic mregDCs after exposure to Gal9+ NPC derived SEVs. Indeed, all the event described above are representative and perfectly in line with what could happen to DCs during their development in the presence of NPC SEVs in the tumour context, and the consequence in term of regulatory gene overexpression (Supplementary data 6 B and D), metabolism shift, and both cytokine release and T cell anergy is strongly in favour of the role of Gal9/TIM3 axis so. Nevertheless, to prove indisputably the role of Gal9 on this process, we decided to move further and use a specific mAb able to block its capacities as we described with our collaborators (Lhuillier et al., 2018; Sarahjibrin, 2021). Our results indicate that this antibody is able to block T cell death or anergy triggered by recombinant Galectine‐9. In the present work, we used this original anti‐Gal9 blocking antibody (1g3) that allows us to prove that the immunosuppressive Gal9 could partly be responsible for the change in the fate of DC in its secreted form but overall associated to extracellular vesicles. This indication reinforces our understanding of the immunosuppressive role played by Gal9 itself in NPC and the ability of EVs to convey this property on target cells.

Finally, in parallel to previous work from the team demonstrating that NPC‐Exo could recruit Tregs at the tumour site both In vitro and in vivo (Mrizak et al., 2015), we intend to demonstrate that NPCSEVs preferentially attract NPCSEV‐DC which could also enhance the suppressive microenvironment present in NPC. Interestingly we observed that tDC do not migrate under any chemotactic gradient as shown in the migration movie of tDC under a C15SEV gradient (Supplementary data 1). This could be explained by the use of vitamin D3 and dexamethasone to generate them. Indeed, another study reports similar results with tDC generated with vitamin D3 or dexamethasone that do not migrate toward a CCL19 gradient and have an under‐expression of CCR7, the CCL19 receptor (Adnan et al., 2016; Barragan et al., 2015). It is important to note that glucocorticoids as dexamethasone are able to cause microtubule and F‐actin rearrangement in T lymphocytes leading to depolarization of the cells preventing their migration (Müller et al., 2013). Same action was described in mesenchymal stem cell that loss migration behaviour after dexamethasone treatment (Schneider et al., 2015) and could have led to the paralytic state of tDC that we observed.

Given that the presence of conventional DCs can be considered very early in tumour development and given the role of mregDCs in resistance to anti‐cancer treatments, it can be assumed that SEVs are one of the main players in this feature. Above immunotherapies, this cellular mechanism could probably explain, at least in part, the fatal resistance observed in too many NPC patients after recurrence of the classical radio‐chemo therapeutic approach.

Altogether our data, offers a new point of view about the role of NPC derived SEV on DCs. This study presents for the first time that tumour derived small extracellular vesicles contribute to NPC immune evasion via the emergence of mregDCs through Gal9. This indirect mechanism enhances the lack of proinflammatory cytokines production and decrease capacity to activate effector T cells probably via IL‐4 production, and thus contribute to the immunosuppressive tumour microenvironment. These results coincide with a single cell study in NPC that described the presence of mregDCs into NPC microenvironment (Chen et al., 2020) and with TCGA dataset in H&N cancer. Tumour derived small extracellular vesicles are major players in tumorigenesis and of great interest as therapeutic targets then. We are now one‐step closer to decipher the immune evasion mechanisms put in place by the tumour although there are many others still to discover. At this time of great promise for anticancer immunotherapies, better understanding tumour immune evasion will help us to improved immunotherapy and develop new immunotherapeutic drugs.

## AUTHOR CONTRIBUTIONS

**Anthony Lefebvre**: Formal analysis; investigation; methodology; software; writing—original draft; writing—review and editing. **Camille Trioën**: Formal analysis; investigation; methodology; writing—original draft; writing—review and editing. **Sarah Renaud**: Investigation; methodology; writing—original draft. **William Laine**: Investigation. **Benjamin Hennart**: Formal analysis; methodology; resources; software. **Clément Bouchez**: Investigation. **Bertrand Leroux**: Investigation; methodology. **Delphine Allorge**: Methodology; resources. **Jérôme Kluza**: Methodology; resources; validation. **Guillaume Paul Grolez**: Formal analysis; investigation. **Nadira Delhem**: Conceptualization; funding acquisition; project administration; validation; writing—review and editing

## CONFLICT OF INTEREST STATEMENT

The authors declare no conflict of interest.

## Supporting information

Supporting Information

Supplementary data 1. Migration of differential moDCs over time after displacement. Representative movies of mDC, tDC and C15SEV‐DC migration under C15SEV gradient over time after displacement analysis using Imaris software.

Supplementary data 2. Monitoring of mouse mass and tumour volume over time. (A) Representative graph of mouse weight monitoring during the growth of C15 NPC cells (*n* = 16). (B) Representative graph of tumour volume after measurement with a caliper over time (*n* = 16).

Supplementary data 3. Representative gating strategies and phenotypical analysis of monocytes and dendritic cells after differentiation and maturation. (A) Representative gating strategy of monocytes and dendritic cells. After forward and side scatter gating and doublet exclusion, cells were analysed for the expression of CD11c and CD14. For marked monocytes, gate was set on CD11c+ CD14+ cells. Then for dendritic cells gate was set on CD11c+ CD14‐ cells. (B) Representative gating strategy of dendritic cells. After forward and side scatter gating and doublet exclusion, cells were analysed for the expression of CD11c and CD14. Gate was set on CD11c‐ CD14‐ cells for unlabelled cells, on CD11c+ CD14‐ for labelled DCs and on CD11+ CD14+ for labelled tolerogenic DCs (tDC).

Supporting Information

Supplementary data 4. Characterization of control monocytes derived dendritic cells after differentiation and maturation. (A) Representative dot plot of monocytes and immature dendritic cells (iDC) after differentiation state for the expression of CD11c and CD14 (A.a) and CD11c and DC‐SIGN (A.b). (B and C) Representative dot plot of iDC (Blue) VS mature dendritic cells (mDC, Red) and mDC (Red) VS tolerogenic DC (tDC, orange), respectively, after maturation process for the expression of CD11c and CD14 (a), CD40 (b), CD80 (c), CD83 (d), CD86 (e), HLA‐DR (f), DC‐SIGN (g).

Supplementary data 5: Control of CD3TL and DC proliferation without coculture in the MLR tests presented in Figure 2B and C. (A) Proliferation assay of CD3 T Lymphocytes (CD3TL) co‐cultured with iDC (iDC/CD3TL), non‐activated CD3TL (NA CD3TL), activated CD3TL (A CD3TL), monocytes and moDCs (iDC, tDC and C15SEV‐iDC) for 48 h (a), 72 h (b) and 96 h (c). Results are expressed in proliferation index normalized on iDC/CD3TL proliferation from three independent experiments, MEAN ± SEM. Statistical differences between conditions were analysed by one‐way ANOVA with *p < 0.05 considered as significant, ***p < 0.001, ****p < 0.0001. (B) Proliferation assay of CD3 T Lymphocytes (CD3TL) co‐cultured with mDC (mDC/CD3TL), non‐activated CD3TL (NA CD3TL), activated CD3TL (A CD3TL) and moDCs (iDC, mDC, tDC and C15SEV‐DC) for 48 h (a), 72 h (b) and 96 h (c). Results are expressed in proliferation index normalized on mDC/CD3TL proliferation from seven (48 and 72 h) and six (96 h) independent experiments, MEAN ± SEM. Statistical differences between conditions were analysed by one‐way ANOVA with *p < 0.05 considered as significant, ***p < 0.001, ****p < 0.0001.

Supplementary data 6. Expression in moDCs of gene associated with maturation or regulation pathway. (A and C) Heat map representing gene expression levels of genes associated with maturation (A) or regulation (C) properties in all moDCs types (mDC, tDC and C15SEV‐DC) from four different donors. Heat map are express in log2(2^‐ΔΔCT) normalized on control mDC. Overexpressed genes are representing in green while under‐expressed genes appear in red as indicated by the scale at the right from the hat map. (B and D) Histogram representing gene expression in 2^^‐ΔΔCT of genes associated with maturation (B) or regulation (D) properties in all moDCs types (mDC, tDC and C15SEV‐DC). The data are representative of four independent experiments normalized on gene expression in control mDC, MEAN ± SEM. Statistical differences were analysed by One Way ANOVA. *p < 0.05, **p < 0.01, ***p < 0.001, ****p < 0.0001.

Supplementary data 7. IL4I1 expression is increased in Dendritic cells in Head and Neck cancer. (A) Relative mRNA levels of IL4I1 in Head and Neck (h&n) cancer and a normal tissue. The whisker boxplots were generated with GEPIA from The Cancer Genome Atlas (TCGA) and Genome Tissue Expression (GTEX) datasets. Relative levels are expressed as log2 transcripts per million bases (TPM). Statistical analyses were performed using an unpaired t‐test (* = p < 0.05). (B) Correlation analysis of IL4I1 and immune infiltrating cell population in H&N cancer TCGA dataset. (partial.cor is for partial correlation coefficient, p is for p‐value). Relative levels are expressed as log2 transcripts per million bases (TPM). (C) Kaplan‐Meier curves assessing the correlation in between the expression level of IL4I1 and the Overall Survival (Left panel) or Relapse free survival (right panel) of H&N tumour patients from TCGA.

Supplementary Table 1. Information on tumour, cells numbers and extracted extracellular vesicles. This table provides information on tumour mass after removal, the numbers of cells after tumour digestion, the amount of supernatant recovered for SEVs isolation, and the protein and particle concentration of SEVs after isolation.
Supplementary Material and Methods .
Analysis of IL4I1 in H&N cancer related datasets
Analyses of relative expression of IL4I1 were conducted using GEPIA tool (Lánczky & Győrffy, 2021) (http://gepia.cancer‐pku.cn/, accessed the 20 december 2022) that integrates a normal tissues from GTEx (*n* = 44) and head and neck cancer dataset from TCGA (*n* = 519). Expression of IL4I1 in different immune cell types were analyzed using TIMER2.0 (B. Li et al., 2016) (http://timer.cistrome.org/, accessed the 20 december 2022). Overall or Relapse Free Survival curves were conducted using Km‐plot tool (Nagy et al., 2021) (https://kmplot.com/analysis/, accessed the 20 december 2022). Quantitative comparisons of the cell proportions or expression in different cell types were analyzed using built‐in t test (GEPIA) or ANOVA test (TIMER2.0).

Supporting Information

Supporting Information

Supporting Information
