# Supplementary figures and images for: Extracellular vesicles derived from nasopharyngeal carcinoma induce the emergence of mature regulatory dendritic cells using a galectin‐9 dependent mechanism
Source: J Extracell Vesicles. 2023 Dec 20;12(12):12390. doi: 10.1002/jev2.12390 (PMC10731827; doi:10.1002/jev2.12390)

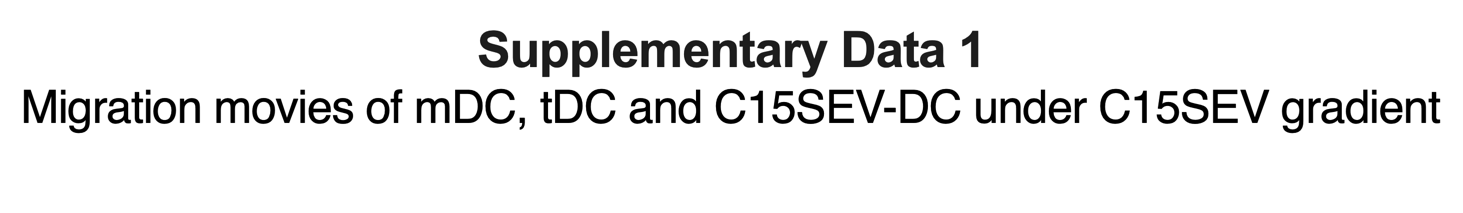

Supplement: Supplementary file 2 — Supplementary data 1. Migration of differential moDCs over time after displacement. Representative movies of mDC, tDC and C15SEV‐DC migration under C15SEV gradient over time after displacement analysis using Imaris software. [file JEV2-12-12390-s008.tiff]

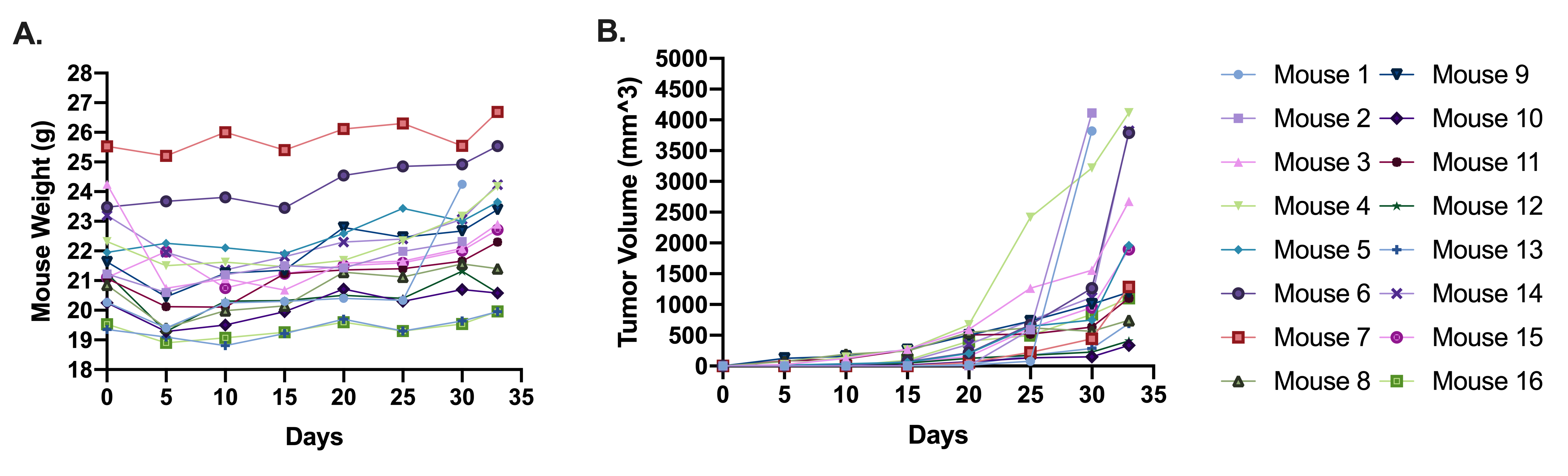

Supplement: Supplementary file 3 — Supplementary data 2. Monitoring of mouse mass and tumour volume over time. (A) Representative graph of mouse weight monitoring during the growth of C15 NPC cells (n = 16). (B) Representative graph of tumour volume after measurement with a caliper over time (n = 16). [file JEV2-12-12390-s012.tiff]

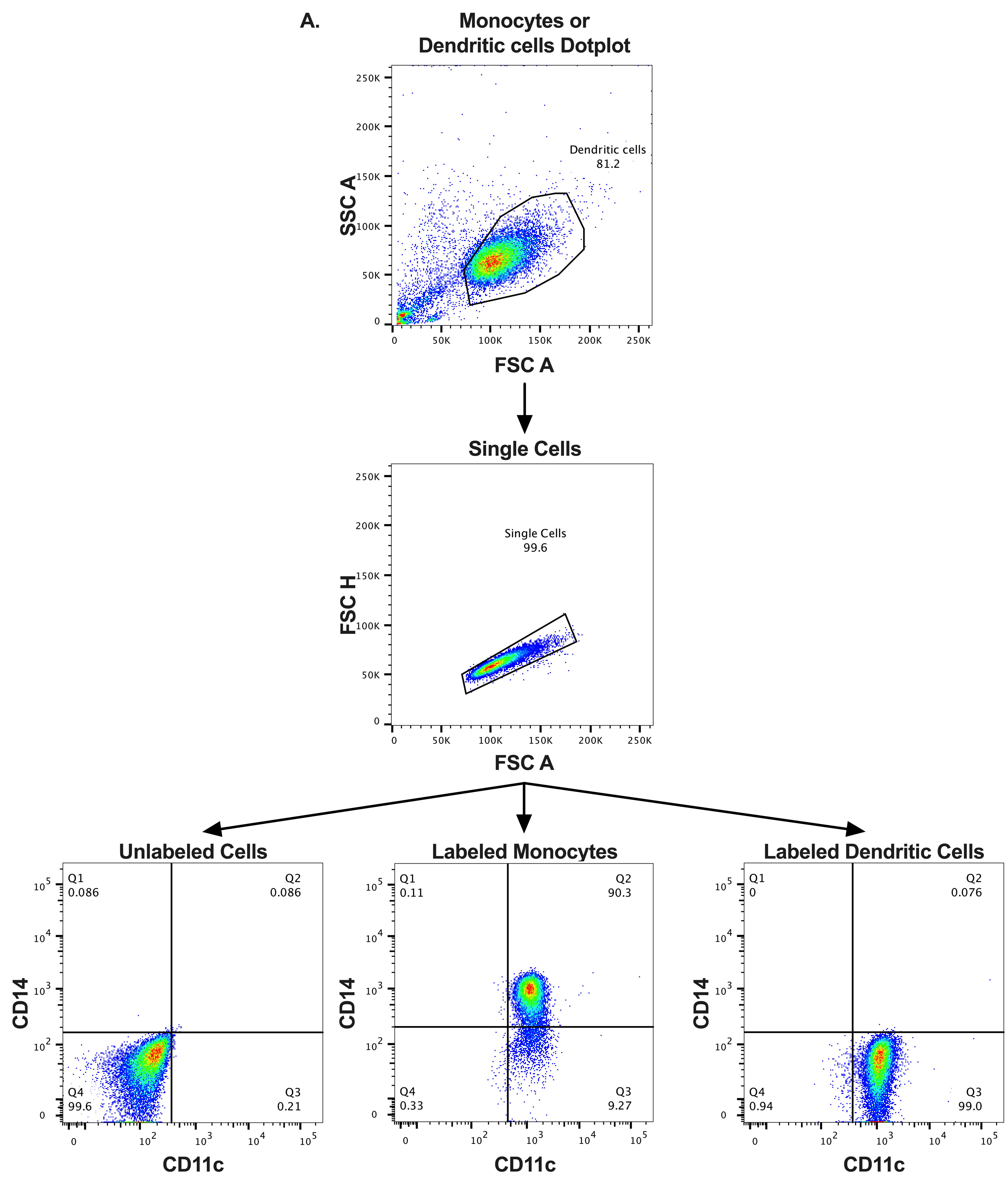

Supplement: Supplementary file 4 — Supplementary data 3. Representative gating strategies and phenotypical analysis of monocytes and dendritic cells after differentiation and maturation. (A) Representative gating strategy of monocytes and dendritic cells. After forward and side scatter gating and doublet exclusion, cells were analysed for the expression of CD11c and CD14. For marked monocytes, gate was set on CD11c+ CD14+ cells. Then for dendritic cells gate was set on CD11c+ CD14‐ cells. (B) Representative gating strategy of dendritic cells. After forward and side scatter gating and doublet exclusion, cells were analysed for the expression of CD11c and CD14. Gate was set on CD11c‐ CD14‐ cells for unlabelled cells, on CD11c+ CD14‐ for labelled DCs and on CD11+ CD14+ for labelled tolerogenic DCs (tDC). [file JEV2-12-12390-s005.tiff]

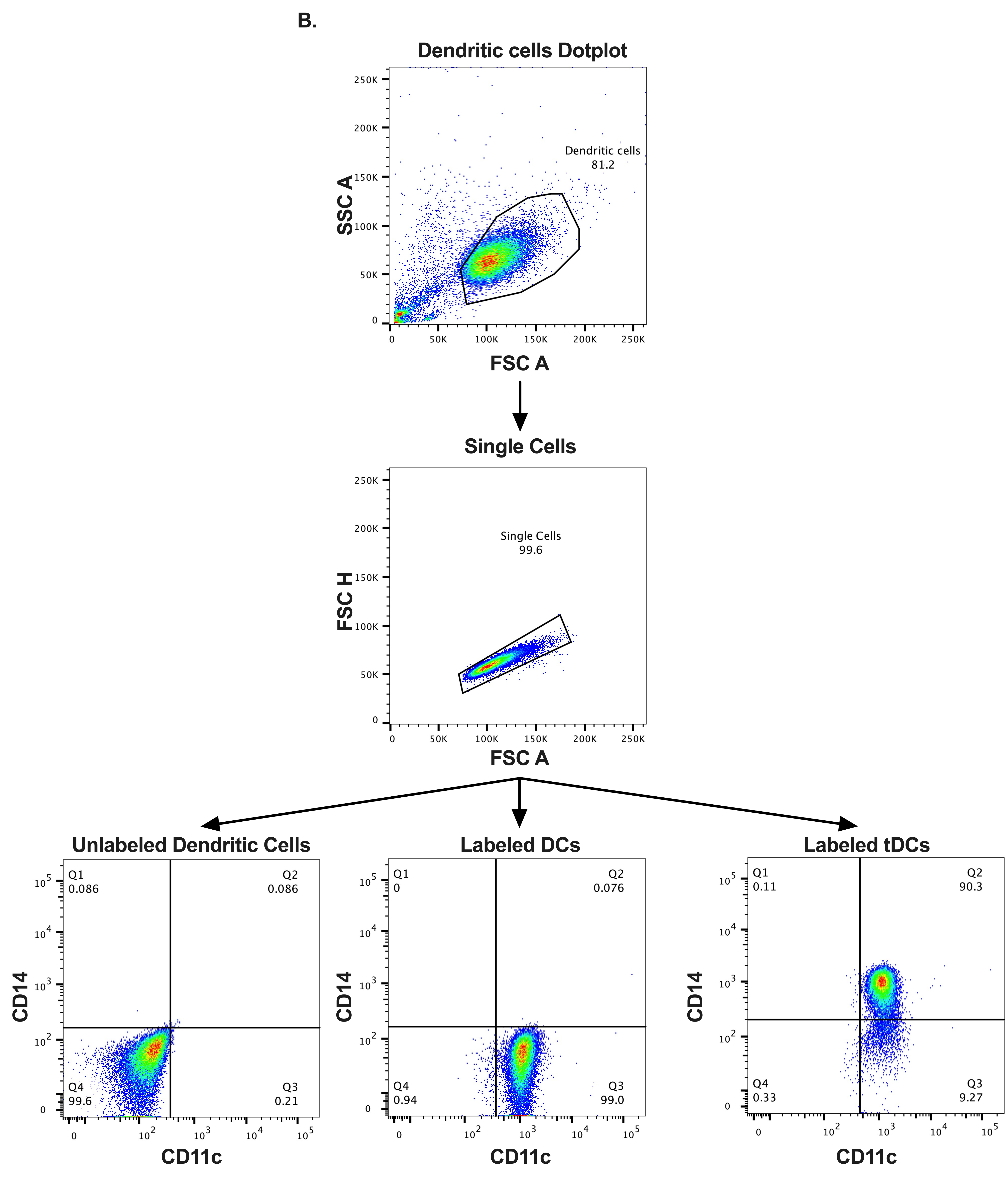

Supplement: Supplementary file 5 — Supporting Information [file JEV2-12-12390-s003.tiff]

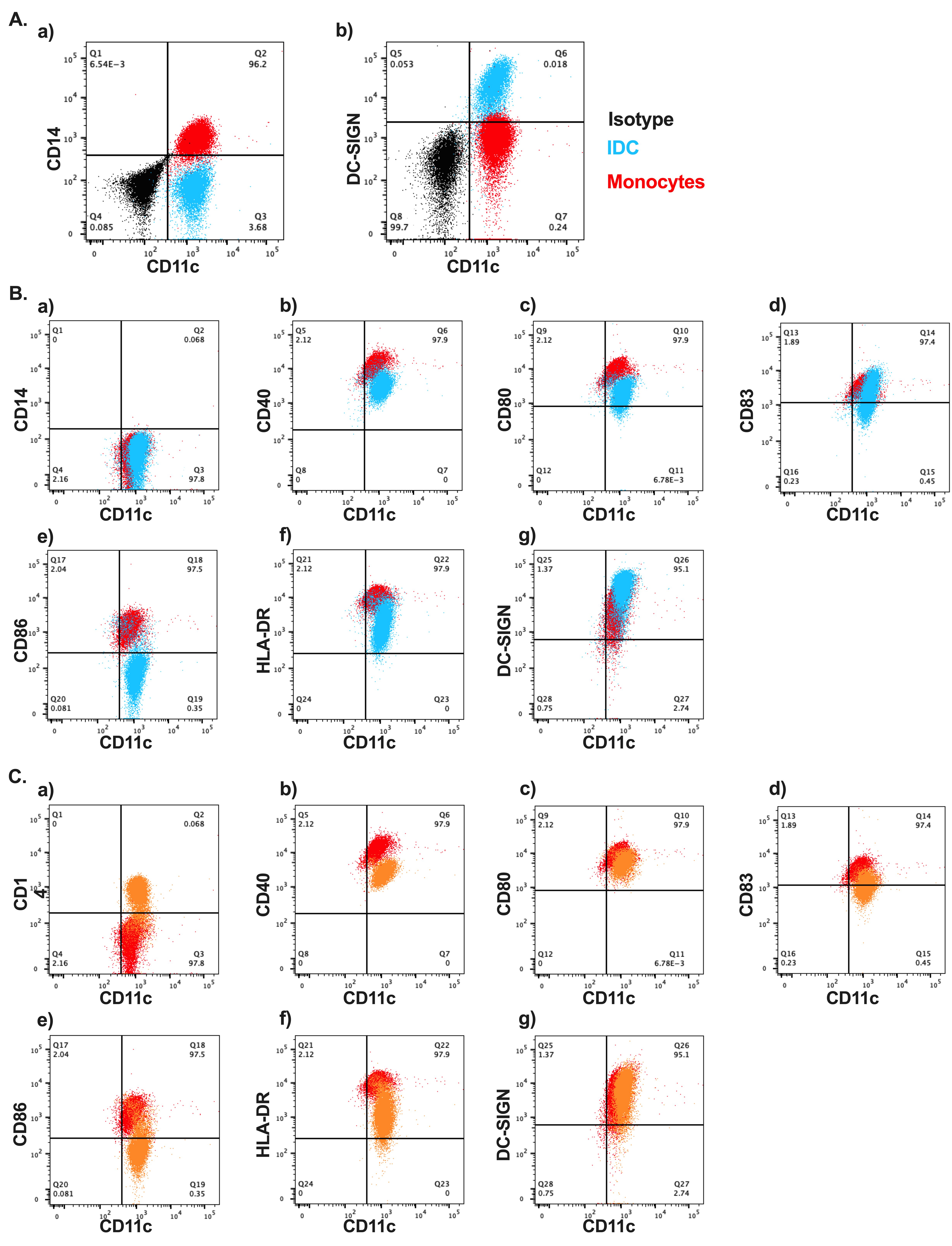

Supplement: Supplementary file 6 — Supplementary data 4. Characterization of control monocytes derived dendritic cells after differentiation and maturation. (A) Representative dot plot of monocytes and immature dendritic cells (iDC) after differentiation state for the expression of CD11c and CD14 (A.a) and CD11c and DC‐SIGN (A.b). (B and C) Representative dot plot of iDC (Blue) VS mature dendritic cells (mDC, Red) and mDC (Red) VS tolerogenic DC (tDC, orange), respectively, after maturation process for the expression of CD11c and CD14 (a), CD40 (b), CD80 (c), CD83 (d), CD86 (e), HLA‐DR (f), DC‐SIGN (g). [file JEV2-12-12390-s004.tiff]

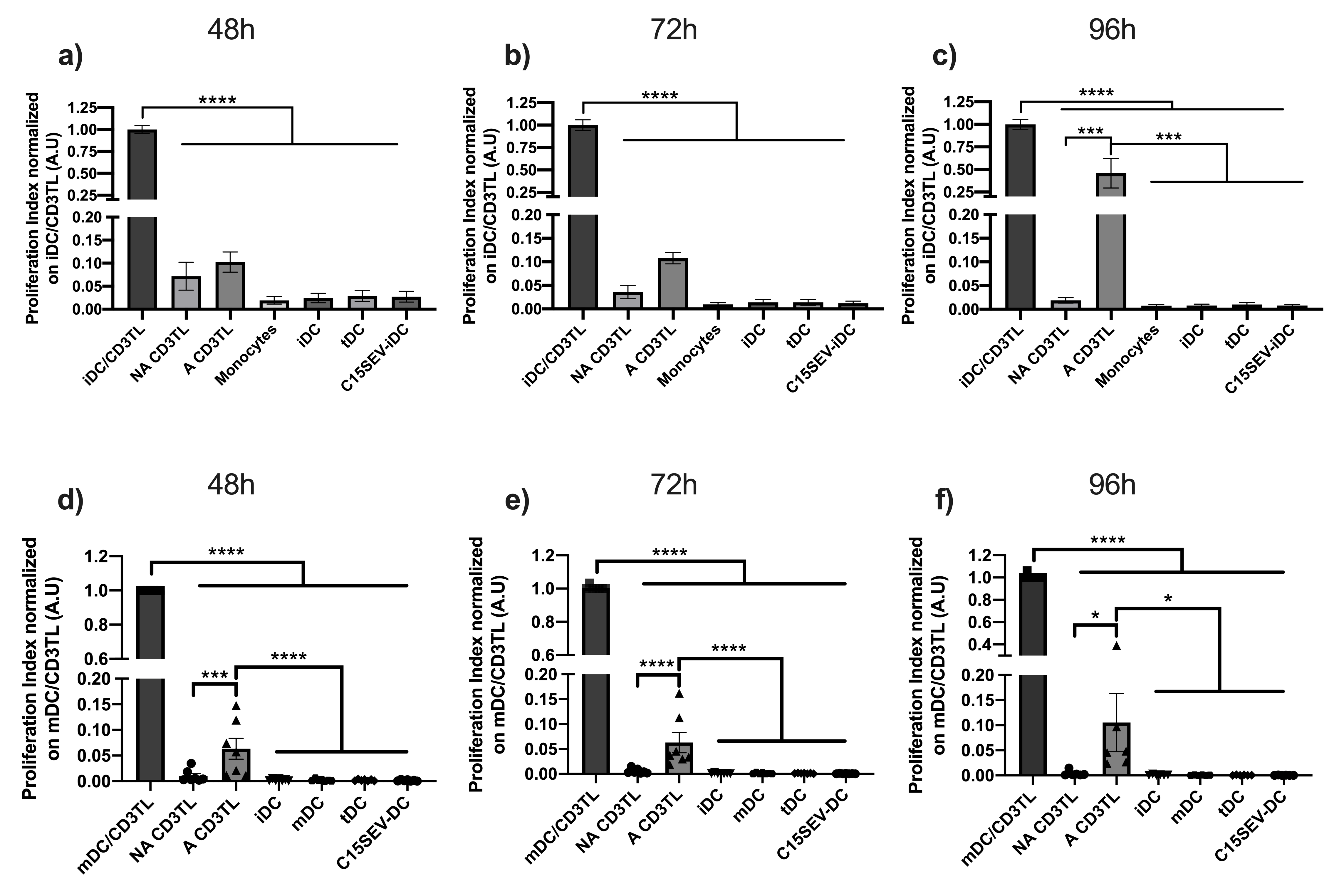

Supplement: Supplementary file 7 — Supplementary data 5: Control of CD3TL and DC proliferation without coculture in the MLR tests presented in Figure 2B and C. (A) Proliferation assay of CD3 T Lymphocytes (CD3TL) co‐cultured with iDC (iDC/CD3TL), non‐activated CD3TL (NA CD3TL), activated CD3TL (A CD3TL), monocytes and moDCs (iDC, tDC and C15SEV‐iDC) for 48 h (a), 72 h (b) and 96 h (c). Results are expressed in proliferation index normalized on iDC/CD3TL proliferation from three independent experiments, MEAN ± SEM. Statistical differences between conditions were analysed by one‐way ANOVA with *p < 0.05 considered as significant, ***p < 0.001, ****p < 0.0001. (B) Proliferation assay of CD3 T Lymphocytes (CD3TL) co‐cultured with mDC (mDC/CD3TL), non‐activated CD3TL (NA CD3TL), activated CD3TL (A CD3TL) and moDCs (iDC, mDC, tDC and C15SEV‐DC) for 48 h (a), 72 h (b) and 96 h (c). Results are expressed in proliferation index normalized on mDC/CD3TL proliferation from seven (48 and 72 h) and six (96 h) independent experiments, MEAN ± SEM. Statistical differences between conditions were analysed by one‐way ANOVA with *p < 0.05 considered as significant, ***p < 0.001, ****p < 0.0001. [file JEV2-12-12390-s002.tiff]

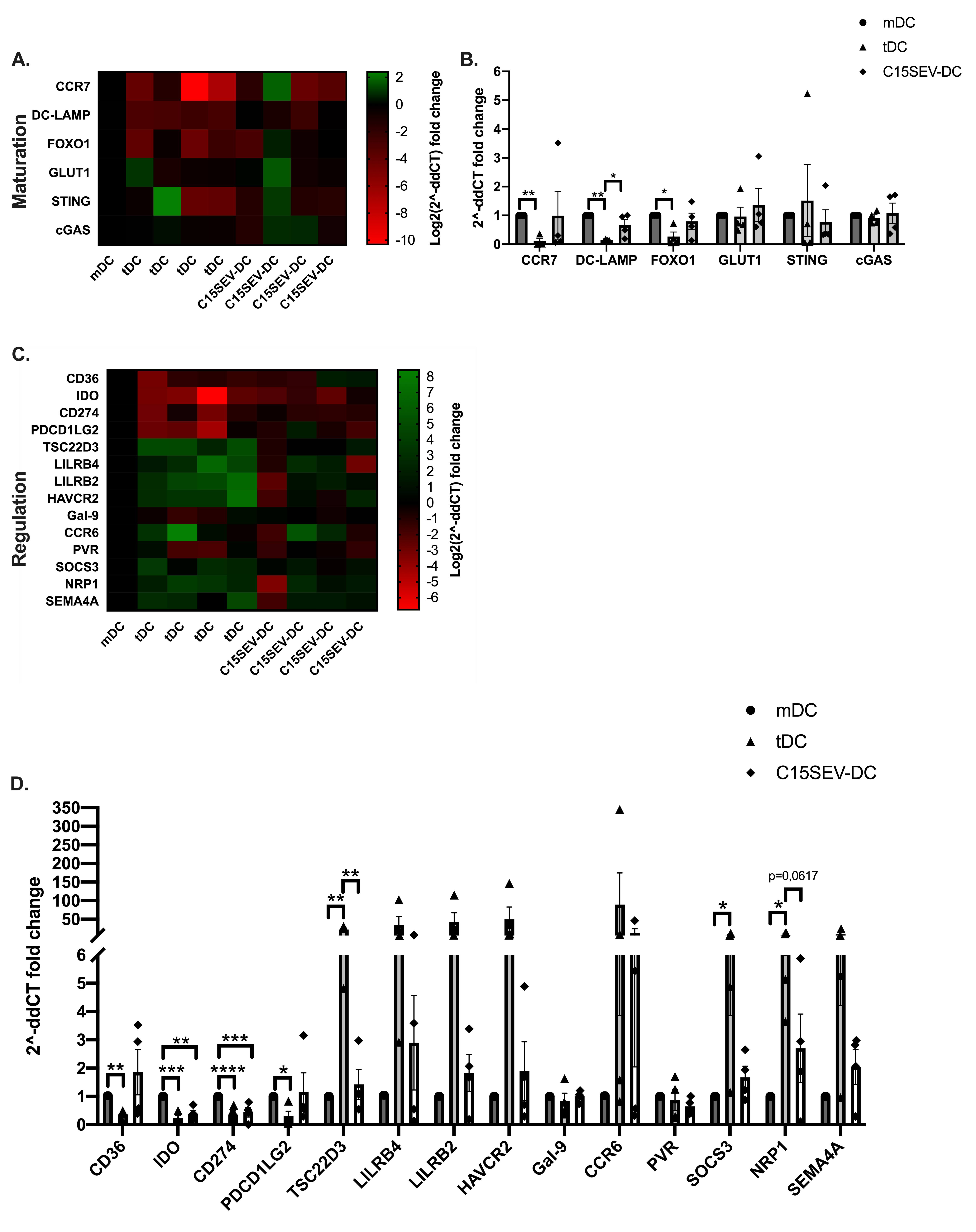

Supplement: Supplementary file 8 — Supplementary data 6. Expression in moDCs of gene associated with maturation or regulation pathway. (A and C) Heat map representing gene expression levels of genes associated with maturation (A) or regulation (C) properties in all moDCs types (mDC, tDC and C15SEV‐DC) from four different donors. Heat map are express in log2(2^‐ΔΔCT) normalized on control mDC. Overexpressed genes are representing in green while under‐expressed genes appear in red as indicated by the scale at the right from the hat map. (B and D) Histogram representing gene expression in 2^^‐ΔΔCT of genes associated with maturation (B) or regulation (D) properties in all moDCs types (mDC, tDC and C15SEV‐DC). The data are representative of four independent experiments normalized on gene expression in control mDC, MEAN ± SEM. Statistical differences were analysed by One Way ANOVA. *p < 0.05, **p < 0.01, ***p < 0.001, ****p < 0.0001. [file JEV2-12-12390-s001.tiff]

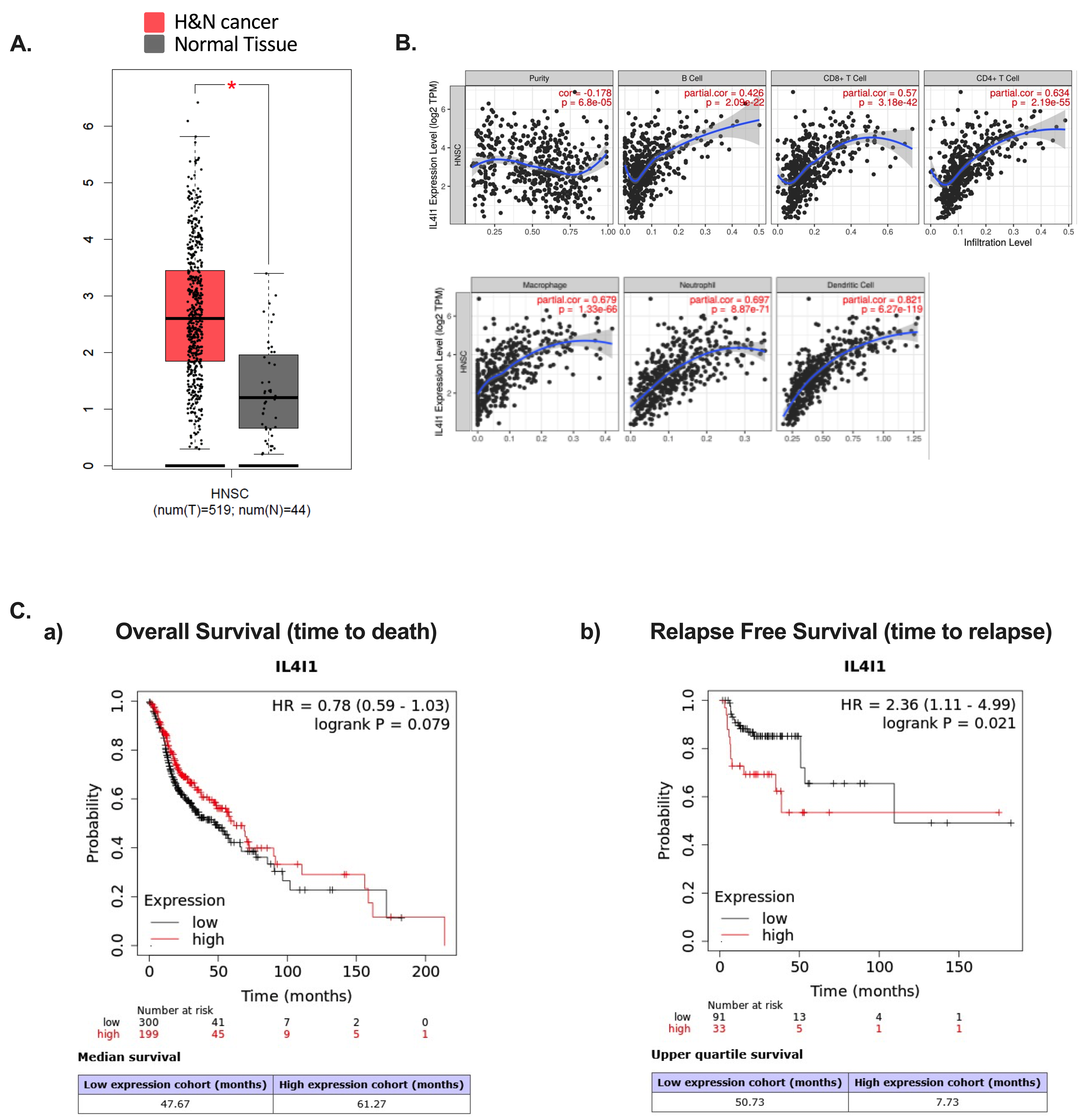

Supplement: Supplementary file 9 — Supplementary data 7. IL4I1 expression is increased in Dendritic cells in Head and Neck cancer. (A) Relative mRNA levels of IL4I1 in Head and Neck (h&n) cancer and a normal tissue. The whisker boxplots were generated with GEPIA from The Cancer Genome Atlas (TCGA) and Genome Tissue Expression (GTEX) datasets. Relative levels are expressed as log2 transcripts per million bases (TPM). Statistical analyses were performed using an unpaired t‐test (* = p < 0.05). (B) Correlation analysis of IL4I1 and immune infiltrating cell population in H&N cancer TCGA dataset. (partial.cor is for partial correlation coefficient, p is for p‐value). Relative levels are expressed as log2 transcripts per million bases (TPM). (C) Kaplan‐Meier curves assessing the correlation in between the expression level of IL4I1 and the Overall Survival (Left panel) or Relapse free survival (right panel) of H&N tumour patients from TCGA. [file JEV2-12-12390-s010.tiff]
