# Supplementary material for: Extracellular vesicles derived from nasopharyngeal carcinoma induce the emergence of mature regulatory dendritic cells using a galectin‐9 dependent mechanism
Source: J Extracell Vesicles. 2023 Dec 20;12(12):12390. doi: 10.1002/jev2.12390 (PMC10731827; doi:10.1002/jev2.12390)
Supplement: Supplementary file 10 — Supplementary Table 1. Information on tumour, cells numbers and extracted extracellular vesicles. This table provides information on tumour mass after removal, the numbers of cells after tumour digestion, the amount of supernatant recovered for SEVs isolation, and the protein and particle concentration of SEVs after isolation. Supplementary Material and Methods . Analysis of IL4I1 in H&N cancer related datasets Analyses of relative expression of IL4I1 were conducted using GEPIA tool (Lánczky & Győrffy, 2021) (http://gepia.cancer‐pku.cn/, accessed the 20 december 2022) that integrates a normal tissues from GTEx (n = 44) and head and neck cancer dataset from TCGA (n = 519). Expression of IL4I1 in different immune cell types were analyzed using TIMER2.0 (B. Li et al., 2016) (http://timer.cistrome.org/, accessed the 20 december 2022). Overall or Relapse Free Survival curves were conducted using Km‐plot tool (Nagy et al., 2021) (https://kmplot.com/analysis/, accessed the 20 december 2022). Quantitative comparisons of the cell proportions or expression in different cell types were analyzed using built‐in t test (GEPIA) or ANOVA test (TIMER2.0). [file JEV2-12-12390-s011.docx]

**Supplementary Table 1. Information on tumour, cells numbers and extracted extracellular vesicles.** This table provides information on tumour mass after removal, the numbers of cells after tumour digestion, the amount of supernatant recovered for SEVs isolation, and the protein and particle concentration of SEVs after isolation.

| **Tumor weight (g)** | **Number of isolated cells** | **Volume of supernatant (mL)** | **SEVs concentration (µg/mL)** | **SEVs concentration (particles/mL)** |
| --- | --- | --- | --- | --- |
| 5.09 ± 1.86 | 1.47E+08 ± 7.46E+07 | 220.5 ± 111.9 | 715.65 ± 229.03 | 5.75E+11 ± 3.75E+11 |
